# Associations between urban exposome and recurrence risk among survivors of acute myocardial infarction in Beijing, China

**DOI:** 10.1016/j.envres.2023.117267

**Published:** 2023-09-28

**Authors:** Ningrui Liu, Qiuju Deng, Piaopiao Hu, Jie Chang, Yan Li, Yuyang Zhang, Yuwei Su, Jing Liu, Ying Long

**Affiliations:** a School of Architecture, Tsinghua University, Beijing, China; b Center for Clinical and Epidemiologic Research, Beijing An Zhen Hospital, Capital Medical University; Beijing Institute of Heart, Lung, and Blood Vessel Diseases; National Clinical Research Center of Cardiovascular Diseases, Beijing, China; c School of Urban Design, Wuhan University, Wuhan, China; d Hang Lung Center for Real Estate, Key Laboratory of Eco Planning & Green Building, Ministry of Education, Tsinghua University, Beijing, China

**Keywords:** Urban exposome, Acute myocardial infarction Prognosis, Recurrence, Urban design, Fine particulate matter

## Abstract

Few previous studies have investigated the impacts of coexposure to multiple urban environmental factors on the prognosis of acute myocardial infarction (AMI) events. This study aimed to evaluate the associations between the urban exposome and AMI recurrence. We used data from 88,509 AMI patients from a large cohort obtained from the Beijing Cardiovascular Disease Surveillance System between 2013 and 2019. Twenty-six types of urban exposures were assessed within 300-m, 500-m, and 1000-m buffers of patients’ home addresses in the baseline and cumulative average levels. We used the Cox proportional hazard model along with the Elastic Net (ENET) algorithm to estimate the hazard ratios (HRs) of recurrent AMI per interquartile range increase in each selected urban exposure. The increased risk of AMI recurrence was significantly associated with lower urban function diversity in the 500-m buffer, longer distance to subway stations and higher PM_2.5_ for both baseline and cumulative average exposure. The cumulative averages of two urban factors, including the distance to parks, and the density of fruit and vegetable shops in the 1000-m buffer, were also identified as significant factors affecting the risk of AMI recurrence. These findings can help improve the urban design for promoting human cardiovascular health.

## 1 Introduction

The burden of ischemic heart disease (IHD) has been increasing steadily in recent years in China, reaching 9.1% of the total burden of disease in 2019 and ranking second among all health outcomes (GBD 2019 Diseases and Injuries Collaborators, 2020). Acute myocardial infarction (AMI) is one of the most severe outcomes in IHD. With the improvement of emergency and in-hospital treatments, majority of AMI patients admitted to hospital are discharged alive. In a nation-wide registry, the in-hospital fatality rate of ST-segment elevation myocardial infarction in China was only 3.1%, 5.3%, and 10.2% in province-level, prefecture-level, and county-level hospitals, respectively (Xu et al., 2020). Although a large number of AMI patients survived at discharge, they were at high risk of recurrence. As people are exposed to urban environments for most of the time during their lifetime, efforts to identify risk factors for urban exposures after initial hospitalization due to AMI events may help mitigate the risk of AMI recurrence.

In this study, we define the urban exposures as the set of built environment, natural spaces, and air pollution, which an individual is exposed to in the outdoor urban environment and can be evaluated through geospatial analysis (Nieuwenhuijsen et al., 2019; Robinson et al., 2018). Previous studies mostly focused on the associations between urban exposures and the incidence or mortality of AMI or IHD, such as associations with building density, green space, distance to main roads, and particulate matters (Griffin et al., 2013; James et al., 2016; Jia et al., 2018; Kan et al., 2008; Mobley et al., 2006; Wang et al., 2019). However, few studies have paid attention to the health effects of various urban exposures on recurrent AMI events (Chen et al., 2020; Liu et al., 2018; Tonne et al., 2016; Zhu et al., 2021). Moreover, most previous studies were limited to one or several urban exposures as the focus based on prior knowledge, whereas individuals are typically exposed to a wide range of urban environmental factors simultaneously.

The concept of the exposome, which encompasses all environmental exposures for an individual from conception to death, was proposed to provide a novel paradigm in environmental health research (Wild, 2012). By considering a broad range of environmental exposures simultaneously, the exposome approach provides the opportunity to comprehensively evaluate the health impacts of various environmental factors. This method can help overcome the shortcomings of traditional hypothesis-based approaches, including the selective reporting of some exposures and the confounding effects of coexposure to multiple environmental factors (Andrianou and Makris, 2018; Siroux et al., 2016). In addition, the exposome approach focuses on the exposure levels in different periods instead of a cross-section of periods. Using the exposome approach, several studies have focused on the effects of multiple prenatal and postnatal urban exposures on various adverse childhood health outcomes, such as low birth weight, obesity, lung functions, and respiratory diseases (Agier et al., 2019; Nieuwenhuijsen et al., 2019; North et al., 2017; Vrijheid et al., 2020). Nevertheless, the exposome approach has been rarely applied to assess the associations between urban environments and cardiovascular risks. Only two relevant studies have studied the effects of urban environments on blood pressure (BP), including one study with a cohort design in Europe and another study with a cross-sectional design in northern China (Song et al., 2022; Warembourg et al., 2021). These researchers found that many significant urban exposures in the single exposure model were not significant when considering multiple exposures, highlighting a disadvantage of the traditional single-exposure approach.

Therefore, this study aimed to systematically evaluate the associations between the urban exposome and AMI recurrence with a cohort design. This study will help deepen the understanding of the health impacts of urban environments on recurrent AMI and provide guidelines for promoting healthier cities.

## 2 Methods

### 2.1 Study design and population

AMI patients were obtained from the Beijing Cardiovascular Disease Surveillance System, which links the records from the Beijing Hospital Discharge Information with the Beijing Vital Registration Monitoring System using a unique personal identity number for Chinese citizens prior to this analysis (Xie et al., 2015). Individuals were eligible for the cohort in this study if they survived in the primary diagnosis of AMI (10th version of the International Classification of Diseases [ICD-10]: I21 for acute myocardial infarction or I22 for subsequent myocardial infarction) between January 1, 2013 and December 31, 2019, and were permanent residents in Beijing. AMI patients who were discharged and rehospitalized on the same day were considered to experience single continuous care episodes (N = 4870). Those patients who were discharged alive within 1 day and without a subsequent readmission on the same day were excluded (N = 963) as they were unlikely to have an AMI. Patients without identifiable home addresses (N = 13,393), without available data of urban exposures and socioeconomic status (N = 7658), and with unclear sex or marital status (N = 1380) were further excluded. Finally, 88,509 AMI patients were included in the final analysis.

### 2.2 Assessment of urban exposome

The spatial coordinates of the home address for all included AMI patients were first geocoded by using the AMAP and Tencent Map API with manual verification (Su et al., 2023). Detailed geocoding procedures are provided in Supporting Information (SI) Section 1. Due to the data availability, it was assumed that the home address of the AMI patient did not change between the first diagnosis and the first recurrence of AMI. The following urban exposure assessment was based on the home address at the first AMI diagnosis. A total of 26 types of candidate urban exposures were selected according to the 5D theory of urban environments (i.e., density, diversity, design, distance to transit, and destination accessibility) and relevant urban exposome studies, which are summarized in Table 1 (Ewing and Cervero, 2010; Nieuwenhuijsen et al., 2019). From the perspective of density, population density, building density, floor area ratio, and urban function density (i. e., the density of points of interest (POIs)) were chosen as the relevant candidate exposures. It should be noted that POIs are virtual geometric points in the online maps (e.g., Google Map, Baidu Map) and represent the location of various urban infrastructures (e.g., shops, parks, schools) in the real world, which are commonly used in geospatial analysis (Psyllidis et al., 2022). The land use diversity and urban function diversity were regarded to represent the aspect of diversity. In addition, several exposures were selected to describe the aspect of design, including road density, distance to main roads, physical disorder, walkability, proportion of green space, green view index, and normalized difference vegetation index (NDVI). The density of subway stations, distance to subway stations, and density of bus stops were considered to represent the distance to transit. The density of a variety of POIs, including parks, tobacco and alcohol retailers, restaurants, fast food restaurants, dessert/drink/pastry shops, vegetable and fruit shops, sport venues, general hospitals, and pharmacies, was taken into account to quantitatively evaluate the destination accessibility. In addition, exposure to fine particulate matters (PM_2.5_) was considered to be associated with cardiovascular diseases (CVDs) in many epidemiological studies (Burnett et al., 2014; GBD 2019 Risk Factors Collaborators, 2020), so PM_2.5_ was also included in the candidate urban exposures. Due to the limit of available data, five candidate exposures (i.e., building density, floor area ratio, physical disorder, walkability, and green view index) were exclusively included in the analysis of patients living in the 5th Ring Road (i.e., urban center) in Beijing, whereas the other 21 candidate exposures were included in the analysis of patients living in all regions of Beijing. Except for those related to distance, all candidate urban exposures were measured within the 300-m, 500-m, and 1000-m buffers, which were approximately equivalent to the 5-min, 10-min, and 15-min walking distances from home, respectively, and usually applied in urban studies (Agier et al., 2019; Nieuwenhuijsen et al., 2019; North et al., 2017; Vrijheid et al., 2020). To reflect the temporal variation in these urban exposures, we first obtained the urban exposures in each year from 2013 to 2019, and then measured the baseline exposure and the cumulative average exposure level (Crouse et al., 2017; Ji et al., 2019). The baseline exposure is defined as the exposure level for patients in the year with the first diagnosis of AMI, and the cumulative average exposure level is defined as the average level from the year with the first diagnosis of AMI to the year with recurrent AMI. Linear interpolation was performed for missing data in corresponding years for urban exposures. For example, for an individual, the first diagnosis and the first recurrence of AMI happened in 2014 and 2018. Then the baseline exposure level refers to the urban exposures in 2014, and the cumulative average exposure level refers to the average urban exposures from 2014 to 2018. Considering the different buffers and exposure assessment methods above, the 26 types of urban exposures can generate 115 variables. Detailed data sources and assessment methods for each urban exposure are provided in SI Section 2. All assessments of urban exposures were conducted using QGIS software (version 3.16.7), and the NDVI measurements were conducted using ENVI software (version 5.3).

### 2.3 Assessment of outcomes

The primary outcome in this study was the first recurrent AMI, and the secondary outcomes were the first fatal and nonfatal recurrent AMI events. Multiple recurrent events of the same individual were not considered in this study. The first recurrent event date for each AMI patient was obtained from the Beijing Cardiovascular Disease Surveillance System. Then, the recurrence interval was calculated from the first discharge date to the first recurrence event date or the end date of the follow-up period (December 31, 2019).

### 2.4 Covariates

Covariates included demographic characteristics, socioeconomic status, and comorbidities. The demographic characteristics included age, sex, and marital status, which were acquired from the Beijing Cardiovascular Disease Surveillance System. The socioeconomic status included the average education years and average annual household income, which was calculated by the estimates in the corresponding traffic analysis zones (TAZs) in Beijing where patients lived (Li et al., 2022). The comorbidities selected included hypertension, hyperlipidemia, diabetes, stroke, heart failure, atrial fibrillation, and renal dysfunction according to other epidemiological studies on the incidence and prognosis of myocardial infarction (Bai et al., 2019; Bally et al., 2017; Bergkvist et al., 2016; Bhaskaran et al., 2011; Chen et al., 2016; Hu et al., 2019; Kojima et al., 2017; Lipsett et al., 2011). These comorbidities were extracted from other discharge diagnosis codes from the Beijing Cardiovascular Disease Surveillance System.

### 2.5 Statistical analysis

When addressing a wide range of complex environmental exposures, variable selection is one of the possible methods to establish the association of exposome with the health outcome in available exposome studies. According to the evaluation methods reported by Agier et al. the performance of several variable selection techniques was compared with each other (Agier et al., 2016). The Elastic Net (ENET) algorithm and DSA (deletion-substitution-addition) algorithm were regarded as the best methods according to their statistical performance assessment, but it is easier to deal with Cox proportional hazard model with ENET algorithm. Therefore, ENET algorithm was selected for use in the subsequent analysis. Details are provided in SI Section 3. The parameters in the ENET algorithm included the mixing proportion for the LASSO (least absolute shrinkage and selection operator) penalty and the ridge penalty, and the overall penalty parameter. They were determined by applying a 10-fold cross-validation and minimizing the root mean squared error (RMSE). In order to avoid overfitting, the optimal parameters were obtained from the sparsest model among those yielding an RMSE within 1 standard error of the minimum RMSE. The above parameter determination followed the methods in Agier et al. (2016).

To obtain the association with the recurrence risk for AMI patients, we first applied the method of an exposome-wide association study (ExWAS) (Agier et al., 2019; Nieuwenhuijsen et al., 2019; Vrijheid et al., 2020) that considered each candidate exposure independently with a Cox proportional hazard model adjusted by covariates. Urban exposures, with a *p* value less than 0.05 in the ExWAS under the correction of multiple hypothesis testing (i.e., Benjamini and Hochberg procedure), were included in the Cox proportional hazard model simultaneously, where the ENET algorithm was applied to realize the variable selection (Agier et al., 2016). Then, the selected variables of urban exposures were finally included in a multivariate Cox model adjusted by covariates. Hazard ratios (HRs) and corresponding 95% confidence intervals per interquartile range (IQR) increase for each of these selected urban exposures were applied to evaluate the associations between these exposures and the risk of AMI recurrence. The association was considered statistically significant if the two-sided *p* value was less than 0.05 in the final multivariate Cox model. We note that, if the same urban exposure within different buffers was selected by the ENET algorithm, they were not simultaneously included in the final multivariate model, and only the variable with lower *p* value in the ExWAS step was retained. The proportional hazard (PH) assumption was diagnosed for each selected urban exposure through Schoenfeld’s residuals test. If some urban exposure variables did not meet the PH assumption, an extended Cox model was further applied to account for the time-varying effects of these variables by incorporating their interaction with the natural logarithm of time (Baulies et al., 2015; Syn et al., 2021; Therneau et al., 2014; Zhang et al., 2018), which is described in detail in SI Section 4. All statistical analyses above were conducted in R software (version 4.1.0).

## 3 Results

Among 88,509 AMI patients, a total of 12,216 recurrent AMI events (13.8%) occurred among 261,296 person-years of follow-up. For the secondary outcomes, a total of 3762 (4.3%) and 9275 (10.5%) recurrent AMI events occurred among 286,080 and 270,224 person-years of follow-up for fatal and nonfatal AMI recurrence, respectively. Detailed characteristics of AMI patients in Beijing and those living within the 5th Ring Road are listed in Table 2. The median age was 66 years old (IQR: 56-77). A total of 62,391 (70.5%) patients were men, and 80,634 (91.1%) patients were married. The baseline and cumulative average exposure levels of all candidate urban exposures are provided in SI Table S2. The spearman correlation matrix was obtained separately for baseline and cumulative average exposure levels, shown in SI Figure S9. The correlations were larger than 0.6 between urban exposure variables within the category of density or destination accessibility and among the same type of urban exposure measured within different buffers.

**Fig. 1:**
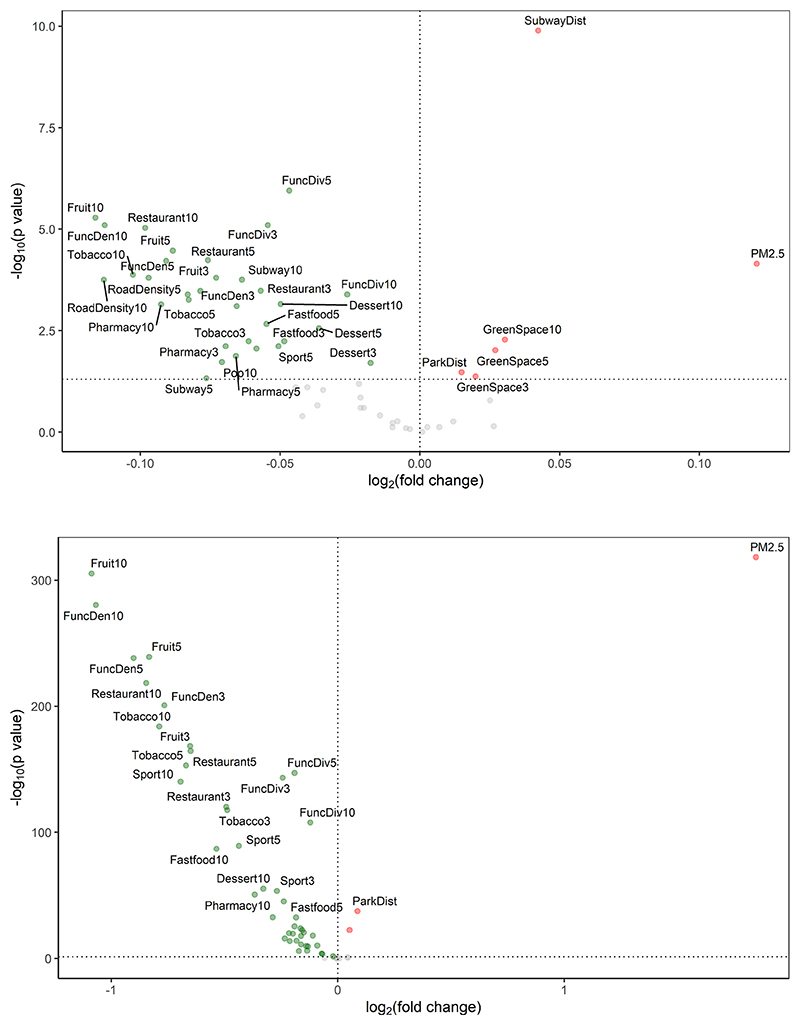
Volcano plot of the coefficient estimates for each urban exposure versus *p* value (adjusted by multiple hypothesis testing, i.e. Benjamini and Hochberg procedure) in the ExWAS analysis. (*a*) Baseline exposure, (*b*) Cumulative average exposure. The grey horizontal dotted line represents the threshold *p =* 0.05. Note: Coefficient estimates are given in the fold change for an IQR change in each variable of urban exposure. The “3”, “5”, and “10” in the labels represent 300-m, 500-m, and 1000-m buffer. Pop = population density, FuncDen = urban function density, FuncDiv = urban function diversity, RoadDensity = road density, GreenSpace = proportion of green space, NDVI = Normalized Difference Vegetation Index, Subway = existence of subway stations, SubwayDist = distance to the nearest subway station, Bus = density of bus stations, ParkDist = distance to parks, Tobacco = density of tobacco and alcohol shops, Restaurant = density of restaurants, Fastfood = density of fast food restaurants, Dessert = density of dessert, drink and pastry shops, Fruit = density of fruit and vegetable shops, Sport = density of sport venues, Pharmacy = density of pharmacies, PM_2.5_ = PM_2.5_ concentration.

Fig. 1 illustrates the results of univariate analysis in the ExWAS. Regarding baseline exposure, 39 variables covering 15 types of urban exposures were statistically significant (*p* < 0.05), among which PM_2.5_, distance to subway stations or parks, and proportion of green space within all three buffers (i.e., 300-m, 500-m, and 1000-m buffers) were significantly associated with increased risk of recurrent AMI. For the cumulative average exposure level, 47 variables covering 16 types of urban exposures were statistically significant. However, a higher concentration of PM_2.5_ and a longer distance to subway stations or parks were significantly associated with higher recurrence risks of AMI events. Detailed results of ExWAS are presented in SI Table S2.

Using the ENET algorithm, eight and five variables of urban exposures were selected for baseline and cumulative average exposure, respectively. Their HRs and 95% CIs for recurrent AMI using the Cox PH model or the extended Cox model are shown in Table 3 and Table 4. The results of testing PH assumption are presented in SI Table S4. We noted that, for those variables meeting the PH assumption, the obtained HRs from these two models were almost the same, and the HRs from extended Cox model were provided in Tables 3 and 4. For those variables not meeting the PH assumption, the HRs from both Cox PH model and extended Cox model were provided in Tables 3 and 4, and the HRs at 1 week, 1 month, 3 months, 6 months, 1 year, 2 years, 3 years, and 5 years were provided for the time-varying effect from the extended Cox model.

Regarding the baseline exposure, higher urban function diversity was significantly associated with a lower risk of AMI recurrence (HR = 0.986, 95% CI: 0.972–0.999). In the Cox proportional hazard model, the increased risk of recurrent AMI was significantly associated with distance to subway stations (HR = 1.030, 95% CI: 1.021–1.040) and PM_2.5_ (HR = 1.136, 95% CI: 1.090–1.184) based on an IQR increase in the baseline exposure level. However, when considering the time-varying effects of these two variables in the extended Cox model, the HR of the distance to subway stations decreased with time and was only significantly associated with AMI recurrence in a short term (within 3 months). In contrast, the HR of PM_2.5_ increased with time and was significantly associated with AMI recurrence in a long term (over 1 month).

Regarding the cumulative average exposure, the risk of AMI recurrence also exhibited significant associations with the distance to urban function diversity within the 500-m buffer (HR = 0.961, 95% CI: 0.949–0.974), subway stations (HR = 1.115, 95% CI: 1.106–1.124), as well as PM_2.5_ (HR = 3.743, 95% CI: 3.643–3.845). Two additional significant urban exposures were identified, including distance to parks (HR = 1.045, 95% CI: 1.032–1.057), and density of fruit and vegetable shops within the 1000-m buffer (HR = 0.665, 95% CI: 0.636–0.696). Except for distance to parks, other four selected urban exposure variables did not meet the PH assumption. However, their time-varying HRs changed slowly with time and were close to the HRs obtained from Cox PH model, except the significant increasing effect for PM_2.5_. Altogether, many significant urban exposures in the ExWAS were not significant in the ENET algorithm.

The associations with fatal and nonfatal recurrent AMI were further examined in this study, as shown in Tables 3 and 4. For the baseline exposure, distance to subway stations were significant risk factors for both fatal and nonfatal recurrent AMI in the short term, which is consistent with results for all recurrent AMI events. However, the time-varying HR of PM_2.5_ was only significant for fatal recurrent AMI but not significant for nonfatal events. The urban function diversity within the 500-m buffer was associated with a decreased risk for fatal recurrent AMI events (HR = 0.945, 95% CI: 0.923–0.967). Regarding the cumulative average exposure scenario, the fatal recurrent AMI risk was significantly associated with urban function diversity within the 500-m buffer, distance to subway stations within one month, and PM_2.5_, whereas the nonfatal recurrent AMI risk was significantly associated with distance to subway stations within three months, and PM_2.5_.

To explore the confounding effects of five urban exposures available only within the 5th Ring Road (i.e., building density, floor area ratio, physical disorder, walkability, and green view index), the associations between urban exposome and recurrent AMI were also estimated among patients living in the 5th Ring Road in Beijing, as shown in SI Table S5. The corresponding results of testing PH assumption are shown in SI Table S6 and Table S7. These five urban exposures were not identified by the ENET algorithm and were not included in the multivariate Cox model. Thus, sufficient evidence was not available to suggest the effect of these five exposures on recurrent AMI events. In addition, as a robustness test, the significant urban exposures identified in this subgroup were compared to those in the entire cohort. For baseline exposure, distance to subway stations, which was significant in the entire cohort, was no longer identified in the subgroup living in the 5th Ring Road, and only PM_2.5_ remained significantly associated with fatal recurrent AMI in this subgroup. For cumulative average exposure, only PM_2.5_ was still identified as a significant risk factor for recurrent AMI events, whereas the other four significant urban exposures in the entire cohort were not identified as significant risk factors in the subgroup within the 5th Ring Road.

## 4 Discussion

### 4.1 Summary of main findings

To the best of our knowledge, this retrospective cohort study is the first study to comprehensively evaluate the associations between recurrent AMI events and the urban exposome. Our results suggest that higher baseline exposure levels of the distance to subway stations and PM_2.5_ and lower baseline exposure levels of urban function diversity within the 500-m buffer are significantly associated with the increased risk of recurrent AMI. We also observed that the risk of recurrent AMI can be significantly increased with higher cumulative average exposures to the distance to parks, the distance to subway stations, and PM_2.5_ and with lower cumulative average exposures to urban function diversity within the 500-m buffer and lower density of fruit and vegetable shops within the 1000-m buffer.

### 4.2 Comparison with previous studies

The finding of a significantly positive association for PM_2.5_ in this study is consistent with previous relevant epidemiological studies. A higher PM_2.5_ concentration was significantly associated with higher risk of hospital readmissions for ST-elevation myocardial infarction or fatal recurrent AMI events in several observational studies (Chen et al., 2016; Liu et al., 2018). Zhu et al. also presented a significant relationship between ambient fine particulate matters and post-MI mortality (pooled HR = 1.07, 95% CI: 1.04–1.09, per 10 μg/m^3^) through a meta-analysis, and explained that the majority of post-MI mortality can be attributable to cardiovascular diseases (Zhu et al., 2021). These associations can be further supported by toxicological evidence which indicates that AMI patients may be sensitive to PM_2.5_ exposure through mechanisms involving systematic oxidative stress and inflammation, atherosclerosis, heart-rate variability, and blood coagulability (Brook et al., 2010; Sacks et al., 2011). Hence, reducing exposure to PM_2.5_ may decrease the risk of recurrent AMI events.

The other four significant urban exposures are related to urban and transport design interventions, including the distance to subway stations, urban function diversity, the distance to parks, and density of fruit and vegetable shops. Giles-Corti et al. noted that urban and transport design interventions indirectly influence health outcomes via transport modes, daily living outcomes, and subsequent risk exposures, such as air pollution, noise, physical inactivity, and unhealthy diet (Giles-Corti et al., 2016, 2022). Nieuwenhuijsen indicated that urban design can affect the walking and other transportation behaviors, which is associated with cardiometabolic risk factors (such as obesity and blood pressure), physical activity, and the exposure to natural environment (such as air and noise pollution), thus further affecting the cardiovascular health (Nieuwenhuijsen, 2018). Detailed discussion about health effects of each of these exposures and possible pathways are conducted below, respectively.

This study reveals that a shorter distance to subway stations was associated with a lower risk of recurrent AMI from the view of both baseline and cumulative average exposure levels. Epidemiological studies directly focusing on this association are lacking. Nevertheless, a shorter distance to public transport and greater public transport density are associated with decreasing sitting time and increasing walking behavior as reported in several cross-sectional studies in multiple countries (Cerin et al., 2022; Giles-Corti et al., 2016; Sallis et al., 2016). Meanwhile, high physical activity was associated with a lower risk of CVD, and physical inactivity accounted for 4.16% of the burden of disease of IHD globally in 2019 (Dinu et al., 2019; GBD, 2019a Risk Factors Collaborators, 2020; Lear et al., 2017). These studies may help explain the association between the distance to subway stations and AMI recurrence risk.

In addition to the distance to subway stations, urban function diversity was also identified as a significant protective factor for recurrent AMI in this study. Urban function diversity measures the function diversity from the view of POIs, whereas land-use mix measures it from the view of land use. Although relevant studies on urban function diversity are still lacking, several studies focusing on compact cities have noted that promoting mixed land use could lower the disease burden of CVD (Shen and Lung, 2020; Stevenson et al., 2016). It has been pointed out that mixed-use urban development can incorporate various functions, such as residential and commercial land uses, in the nearby neighborhood and promote walking for transport, which can facilitate physical activity and prevent non-communicable diseases such as CVD (Giles-Corti et al., 2016, 2022). More studies are needed in the future to explore the possible mechanism and strengthen the evidence on the influence of urban function diversity on AMI.

Besides, this study identified the distance to parks as a significant exposure that might reduce the risk of recurrent AMI. Although little attention has been given to associations between parks and recurrent AMI, several cohort studies revealed that living nearby parks or a higher proportion of parks was significantly associated with lower risks of AMI or general CVD (Chen et al., 2020; Seo et al., 2019; Tamosiunas et al., 2014). Only one cross-sectional study obtained a negative health impact of access to parks on myocardial infarction, which may be attributable to the weak causal strength of the cross-sectional design (Chum and O’Campo, 2015). Parks may influence the physical activity of individuals and mitigate air and noise pollution, which are all risk factors for IHD or, generally, CVD (Diener and Mudu, 2021; Giles-Corti et al., 2016; Margaritis and Kang, 2017). The findings in this study support the positive health effect of parks on cardiovascular health.

Finally, the density of fruit and vegetable shops within the 1000-m buffer was recognized as a protective factor against recurrent AMI. Giles-Corti et al. suggested that these shops affected health outcomes through a mechanism involving daily diets (Giles-Corti et al., 2016, 2022). Supermarket proximity is significantly positively associated with the intake of fruit and vegetables by cross-sectional studies in Western countries (Michimi and Wimberly, 2010; Thornton et al., 2012). A diet low in fruit and vegetables was significantly associated with an increased risk of IHD based on a meta-analysis, and accounted for 5.62% and 4.61% of the disease burden of IHD worldwide, respectively (Gan et al., 2015; GBD, 2019a Risk Factors Collaborators, 2020). The results in this study are consistent with previous perspectives and advocate more fruit and vegetable shops within individuals’ walkable regions.

### 4.3 Strengths and limitations

This study has several strengths. First, this study focused on the association between urban exposures and recurrent AMI events, whereas previous studies paid more attention to the associations with incidence of AMI events instead of their prognosis. Second, this study employed a retrospective cohort design, which can strengthen the causal association between urban exposures and recurrent AMI events compared to the cross-sectional design for many existing urban studies. Third, this study referred to the statistical methods in previous exposome studies, which first include as many urban exposures as possible and applied variable selection techniques to identify important exposures. Compared to the traditional hypothesis-based approach focusing on one or several exposures, this approach can consider the confounding effects among various urban exposures and avoid selecting false exposures or omitting possible important exposures. As shown in this study, many significant urban exposures in the traditional single-exposure approach were not significant in the exposome analysis, suggesting that the traditional hypothesis-based model may result in a high false discovery proportion (FDP) of the exposures. Fourth, to reflect the temporal dynamics of the exposure, this study evaluated the baseline exposure level and the cumulative average exposure level and explored the association of recurrent AMI with urban exposures on these two different time scales. The findings of this study can lay the foundation for understanding the burden of AMI attributable to urban exposures, and help improve the urban design guidelines for healthier cities and bring potential health benefits to urban populations.

There are still some limitations in this study. First, this study did not consider several potential confounders, such as the use of medications, important behavioral factors, psychosocial factors, and lifestyle factors (such as smoking and BMI) that may affect prognosis after AMI, due to the lack of data. However, we have adjusted for many important demographics, comorbidities, and SES in the statistical analysis. To be noted, the SES data included in the statistical analysis were at the TAZ level instead of the individual level, due to data availability, which is also a limitation. Second, some AMI patients without available data of urban exposures and socioeconomic status were excluded in this study, while these patients may live in the most remote townships in suburban Beijing. Therefore, selection bias may exist and influence the associations between urban exposures and recurrent AMI. Third, in addition to various urban exposures, people are simultaneously co-exposed to many other environmental factors, such as indoor environmental risks (e.g., indoor air pollution and heating) and outdoor meteorological factors (e. g., temperature and ultraviolet radiation). The high-resolution historical data of some factors above are not available for this cohort, especially for indoor environmental risks. Although this study has considered as many urban exposures as possible, future research can conduct a more comprehensive survey of environmental exposures and adjust the health effect of urban exposures obtained in this study. Fourth, it is assumed that the home address of the AMI patient did not change during the follow-up, which may influence the urban exposure assessment. Fifth, more exposure assessment methods can be adopted, such as the maximum exposure level during the follow-up, to explore more associations between urban exposures and AMI recurrence.

## 5 Conclusions

This retrospective cohort study is, to our knowledge, the first to comprehensively evaluate the associations of the urban exposome with recurrent AMI events. It reveals that risk of AMI recurrence might be associated with several urban exposures, including PM_2.5_, the distance to subway stations, urban function diversity, the distance to parks, and density of fruit and vegetable shops. These findings indicate that preventive measures, such as reducing PM_2.5_ concentrations, increasing the urban function diversity and density of fruit and vegetable shops, and reducing the distance to subway stations and parks, can be considered in urban policies and urban design guidelines, which may aid in reducing the recurrent AMI events and bring potential health benefits to urban populations.

## Supplementary Material

**Appendix A. Supplementary data**

Supplementary data to this article can be found online at https://doi.org/10.1016/j.envres.2023.117267

SI

## Figures and Tables

**Table 1:** Candidate urban exposures in this study.

	Urban exposure	Data availability
1.Density	Population density	All regions in Beijing
	Building density	5th Ring Road in Beijing
	Floor area ratio	5th Ring Road in Beijing
	Urban function density	All regions in Beijing
2.Diversity	Land use diversity	All regions in Beijing
	Urban function diversity	All regions in Beijing
3.Design	Road density	All regions in Beijing
	Distance to main roads	All regions in Beijing
	Physical disorder	5th Ring Road in Beijing
	Walkability	5th Ring Road in Beijing
	Proportion of green space	All regions in Beijing
	Green view index	5th Ring Road in Beijing
	NDVI	All regions in Beijing
4.Distance to transit	Density of/Distance to subway stations	All regions in Beijing
	Density of bus stops	All regions in Beijing
5.Destination accessibility	Density of/Distance to parks	All regions in Beijing
	Distance to large green space	All regions in Beijing
	Density of tobacco and alcohol retailers	All regions in Beijing
	Density of restaurants	All regions in Beijing
	Density of fast food restaurants	All regions in Beijing
	Density of dessert/drink/pastry shops	All regions in Beijing
	Density of fruit and vegetable shops	All regions in Beijing
	Density of sport venues	All regions in Beijing
	Density of/Distance to general hospitals	All regions in Beijing
	Density of pharmacies	All regions in Beijing
6.Others	Annual PM_2,5_ concentration	All regions in Beijing

**Table 2:** Characteristics of the AMI patients from 2013 to 2019.

	All regions in Beijing^a^	5th Ring Road in Beijing^a,b^
Total	88,509 (100%)	43,374 (49.0%)
Age (year)	66 (56–77)	67 (57–78)
Sex		
Male	62,391 (70.5%)	30,821 (71.1%)
Female	26,118 (29.5%)	12,553 (28.9%)
Marital status		
Not married	7875 (8.9%)	3832 (8.8%)
Married	80,634 (91.1%)	39,542 (91.2%)
Average education years (year) ^c^	12.2 (11.2–13.0)	12.7 (12.1–13.2)
Average annual household income (ten thousand RMB) ^c^	9.9 (8.4–11.4)	11.2 (9.9–12.1)
Comorbidities		
Hypertension	60,145 (68.0%)	29,440 (67.9%)
Hyperlipidemia	59,148 (66.8%)	25,497 (58.8%)
Diabetes	35,094 (39.7%)	17,757 (40.9%)
Stroke	21,478 (24.3%)	10,530 (24.3%)
Heart failure	34,079 (38.5%)	17,777 (41.0%)
Atrial fibrillation	8614 (9.7%)	4605 (10.6%)
Renal dysfunction	11,690 (13.2%)	6363 (14.7%)

^a^ Number (%) for categorical data, or median (25th to 75th percentile) for continuous data. ^b^ The percentage for total number is the proportion of the patients in the 5th Ring Road among all patients in Beijing, while the other percentages are the proportion of the subgroup among all patients in the 5th Ring Road. ^c^ Average education years and average annual household income were measured at the level of TAZs, while other characteristics were measured at the individual level.

**Table 3:** Adjusted associations between the baseline exposure level of selected urban exposures and all, fatal, or nonfatal recurrent AMI events for all the cohort.

Variable	Buffer	IQR	All recurrent AMI		Fatal recurrent AMI		Nonfatal recurrent AMI	
			HR (95% CI) ^a^	*p* value	HR (95% CI) ^a^	*p* value	HR (95% CI) ^a^	*p* value
Urban function diversity (—)	500 m	0.314	0.986 (0.972–0.999) b	0.037	0.945 (0.923–0.967) b	<0.001	0.999 (0.983–1.015)	0.887
Road density (km/km^2^)	500 m	5.232	0.970 (0.933–1.008)	0.124	0.988 (0.921–1.060)	0.729	0.960 (0.919–1.004)	0.073
Proportion of green space (—)	1000 m	0.110	1.009 (0.994–1.025)	0.222	1.009 (0.981 –1.037)	0.539	1.012 (0.995–1.030)	0.169
Distance to subway stations (km)	–	3.849	1.030 (1.021–1.040) c	<0.001	1.030 (1.013–1.048) c	<0.001	1.028 (1.018–1.038) c	<0.001
1 week (7 days)			1.052 (1.034–1.070)^b^	<0.001	1.051 (1.012–1.091) b	0.009	1.050 (1.030–1.070) b	<0.001
1 month (30 days)			1.038 (1.018–1.058) b	<0.001	1.037 (0.995–1.081)	0.087	1.036 (1.014–1.059) b	0.001
3 months (90 days)			1.028 (1.006–1.050) b	0.012	1.027 (0.980–1.075)	0.262	1.026 (1.002–1.051) b	0.037
6 months (180 days)			1.022 (0.998–1.046)	0.070	1.020 (0.971–1.072)	0.424	1.020 (0.994–1.046)	0.143
1 year (365 days)			1.015 (0.990–1.041)	0.234	1.014 (0.962–1.069)	0.610	1.013 (0.985–1.042)	0.363
2 years (730 days)			1.009 (0.982–1.036)	0.510	1.008 (0.952–1.066)	0.795	1.007 (0.977–1.037)	0.664
3 years (1095 days)			1.005 (0.978–1.034)	0.705	1.004 (0.947–1.064)	0.898	1.003 (0.972–1.035)	0.854
5 years (1825 days)			1.001 (0.972–1.031)	0.955	0.999 (0.940–1.063)	0.980	0.998 (0.966–1.031)	0.918
Density of fast food restaurants (number)	300 m	1	0.988 (0.965–1.011)	0.306	0.980 (0.939–1.022)	0.348	0.991 (0.965–1.018)	0.500
Density of fruit and vegetable shops (number)	300 m	4	0.996 (0.968–1.025)	0.800	1.014 (0.962–1.069)	0.599	0.988 (0.956–1.021)	0.470
Density of pharmacies (number)	300 m	2	0.994 (0.957–1.033)	0.773	0.969 (0.903–1.040)	0.386	1.020 (0.976–1.065)	0.378
PM_2.5_ (μg/m^3^)	–	32.200	1.136 (1.090–1.184) c	<0.001	1.330 (1.232–1.436) c	<0.001	1.107 (1.056–1.161) c	<0.001
1 week (7 days)			1.074 (0.983–1.174)	0.115	1.117 (0.922–1.354)	0.258	1.070 (0.969–1.182)	0.180
1 month (30 days)			1.116 (1.007–1.236) b	0.036	1.222 (0.983–1.520)	0.071	1.093 (0.975–1.226)	0.127
3 months (90 days)			1.148 (1.023–1.289) b	0.019	1.308 (1.025–1.669) b	0.031	1.111 (0.976–1.264)	0.112
6 months (180 days)			1.169 (1.032–1.325) b	0.014	1.365 (1.051–1.773) b	0.020	1.122 (0.976–1.291)	0.107
1 year (365 days)			1.191 (1.041–1.363) b	0.011	1.426 (1.076–1.889) b	0.013	1.134 (0.974–1.319)	0.104
2 years (730 days)			1.213 (1.049–1.402) b	0.009	1.488 (1.101–2.011) b	0.010	1.145 (0.973–1.348)	0.103
3 years (1095 days)			1.226 (1.053–1.426) b	0.008	1.526 (1.115–2.087) b	0.008	1.152 (0.972–1.365)	0.103
5 years (1825 days)			1.242 (1.059–1.456) b	0.008	1.574 (1.133–2.188) b	0.007	1.161 (0.971 –1.388)	0.103

^a^ Coefficients are provided for an IQR increase in the given variable of urban exposome, adjusted by age, sex, marital status, average education years, average annual household income, and the history of complications (hyperlipidemia, diabetes, stroke, heart failure, atrial fibrillation, COPD, and renal dysfunction). ^b^ *p <* 0.05. ^c^ Coefficients are provided by the Cox proportional hazard model (i.e., constant HRs) instead of the extended model with time-varying coefficients.

**Table 4:** Adjusted associations between the cumulative average exposure level of selected urban exposures and all, fatal, or nonfatal recurrent AMI events for all the cohort.

Variable	Buffer	IQR	All recurrent AMI		Fatal recurrent AMI		Nonfatal recurrent AMI	
			HR (95% CI) ^a^	*p* value	HR (95% CI) ^a^	*p* value	HR (95% CI) ^a^	*p* value
Urban function diversity (—)	500 m	0.226	0.961 (0.949–0.974)	<0.001	0.951 (0.925–0.978) b	<0.001	0.995 (0.978–1.013)	0.594
1 week (7 days)			0.980 (0.955–1.007)	0.143				
1 month (30 days)			0.966 (0.938–0.995) b	0.023				
3 months (90 days)			0.955 (0.924–0.988) b	0.007				
6 months (180 days)			0.948 (0.915–0.983) b	0.004				
1 year (365 days)			0.942 (0.906–0.979) b	0.002				
2 years (730 days)			0.935 (0.897–0.974) b	0.001				
3 years (1095 days)			0.931 (0.892–0.972) b	0.001				
5 years (1825 days)			0.926 (0.885–0.969) b	<0.001				
Distance to subway stations (km)	–	3.323	1.115 (1.106–1.124)	<0.001	1.020 (1.004–1.036)	0.012	1.025 (1.015–1.034)	<0.001
1 week (7 days)			1.122 (1.103–1.141) b	<0.001	1.052 (1.016–1.089) b	0.004	1.044 (1.028–1.061)^b^	<0.001
1 month (30 days)			1.122 (1.100–1.143) b	<0.001	1.035 (0.996–1.076)	0.082	1.031 (1.013–1.049) b	<0.001
3 months (90 days)			1.121 (1.097–1.146) b	<0.001	1.022 (0.979–1.067)	0.313	1.021 (1.001–1.041) b	0.042
6 months (180 days)			1.121 (1.095–1.148) b	<0.001	1.014 (0.969–1.062)	0.54	1.014 (0.993–1.036)	0.184
1 year (365 days)			1.121 (1.093–1.150) b	<0.001	1.006 (0.958–1.057)	0.796	1.008 (0.985–1.031)	0.487
2 years (730 days)			1.121 (1.091–1.152) b	<0.001	0.999 (0.948–1.053)	0.963	1.002 (0.978–1.027)	0.877
3 years (1095 days)			1.121 (1.090–1.153) b	<0.001	0.994 (0.941–1.050)	0.836	0.998 (0.973–1.024)	0.897
5 years (1825 days)			1.121 (1.088–1.155) b	<0.001	0.989 (0.934–1.047)	0.694	0.994 (0.968–1.021)	0.647
Distance to parks (km)	^–^	0.673	1.048 (1.035–1.061) b	<0.001	0.993 (0.969–1.016) ^c^	0.538	0.995 (0.981–1.010)	0.515
1 week (7 days)					1.000 (0.949–1.054)	0.992		
1 month (30 days)					0.997 (0.940–1.057)	0.915		
3 months (90 days)					0.994 (0.932–1.060)	0.859		
6 months (180 days)					0.993 (0.927–1.063)	0.831		
1 year (365 days)					0.991 (0.921–1.066)	0.806		
2 years (730 days)					0.989 (0.915–1.070)	0.786		
3 years (1095 days)					0.988 (0.911–1.072)	0.775		
5 years (1825 days)					0.987 (0.907–1.074)	0.763		
Density of fruit and vegetable shops (number)	1000 m	39	0.665 (0.636–0.696)	<0.001	1.019 (0.955–1.089)	0.565	0.961 (0.922–1.002)	0.059
1 week (7 days)			0.695 (0.630–0.768) b	<0.001				
1 month (30 days)			0.667 (0.596–0.746) b	<0.001				
3 months (90 days)			0.647 (0.570–0.733) b	<0.001				
6 months (180 days)			0.634 (0.554–0.726) b	<0.001				
1 year (365 days)			0.621 (0.537–0.718) b	<0.001				
2 years (730 days)			0.609 (0.521–0.712) b	<0.001				
3 years (1095 days)			0.602 (0.512–0.708) b	<0.001				
5 years (1825 days)			0.593 (0.501–0.704) b	<0.001				
PM_2.5_ (μg/m^3^)	^–^	16.995	3.743 (3.643–3.845)	<0.001	1.148 (1.084–1.217) b	<0.001	1.037 (1.002–1.073) b	0.036
1 week (7 days)			2.892 (2.725–3.068) b	<0.001				
1 month (30 days)			3.672 (3.427–3.935) b	<0.001				
3 months (90 days)			4.399 (4.066–4.758) b	<0.001				
6 months (180 days)			4.929 (4.527–5.367)^b^	<0.001				
1 year (365 days)			5.536 (5.048–6.071) b	<0.001				
2 years (730 days)			6.204 (5.616–6.853) b	<0.001				
3 years (1095 days)			6.631 (5.976–7.357) b	<0.001				
5 years (1825 days)			7.211 (6.463–8.046) b	<0.001				

^a^ Coefficients are provided for an IQR increase in the given variable of urban exposome, adjusted by age, sex, marital status, average education years, average annual household income, and the history of complications (hyperlipidemia, diabetes, stroke, heart failure, atrial fibrillation, COPD, and renal dysfunction). ^b^ *p <* 0.05. ^c^ Coefficients are provided by the Cox proportional hazard model (i.e., constant HRs) instead of the extended model with time-varying coefficients.

## Data Availability

Data will be made available on request.
