# Supplementary material for: Associations between urban exposome and recurrence risk among survivors of acute myocardial infarction in Beijing, China
Source: Environ Res. Author manuscript; Available in PMC 2023 Dec 1. (PMC7615203; doi:10.1016/j.envres.2023.117267)
Supplement: SI [file EMS189122-supplement-SI.docx]

***Supporting Information***

**Associations between urban exposome and recurrence risk among survivors of acute myocardial infarction in Beijing, China**

Ningrui Liu^1^, Qiuju Deng^2^, Piaopiao Hu^2^, Jie Chang^2^, Yan Li^1^, Yuyang Zhang^1^, Yuwei Su^1,3^, Jing Liu^2,*^, Ying Long^1,4,^*

^1^ School of Architecture, Tsinghua University, Beijing, China

^2^ Center for Clinical and Epidemiologic Research, Beijing An Zhen Hospital, Capital Medical University; Beijing Institute of Heart, Lung, and Blood Vessel Diseases; National Clinical Research Center of Cardiovascular Diseases, Beijing, China

^3^ School of Urban Design, Wuhan University, Wuhan, China

^4^ Hang Lung Center for Real Estate, Key Laboratory of Eco Planning & Green Building, Ministry of Education, Tsinghua University, Beijing, China

* Correspondence

Ying Long, Email: ylong@tsinghua.edu.cn

Jing Liu, Email: jingliu@ccmu.edu.cn

This appendix has 38 pages, including 6 sections, 7 tables, and 9 figures.

**Section 1 Geocoding procedure of home addresses**

The detailed geocoding procedure of home addresses is introduced in this section, which is also illustrated in **Figure S1** ([Su et al., 2023](#_ENREF_8)). In the section “study design and population” in the main text, the cohort of AMI patients in Beijing from 2013 to 2019 has been selected out from Beijing Monitoring System for Cardiovascular Diseases (denoted as the System below). The geocoding procedure is based on the home address information which the patients registered at the time of hospital admission.

**Figure S1**. The procedure of geocoding the home addresses for AMI patients.


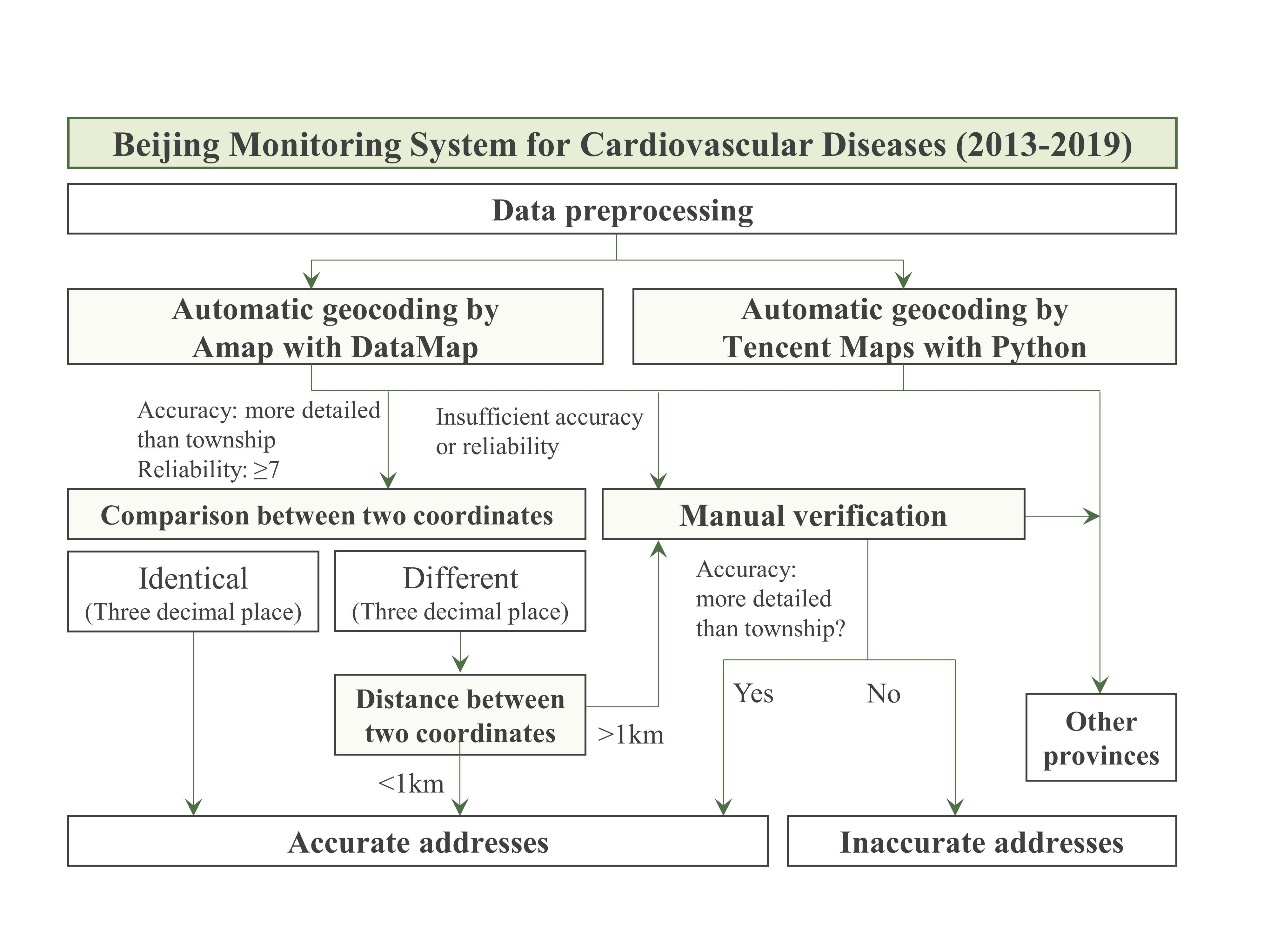


First, the automatic geocoding was conducted via two parallel methods, in order to ensure the accuracy of the geocoding results. On one hand, DataMap software (Version 6.6.2) in the Excel was applied to transform the characters of home addresses into the spatial coordinates. The evaluation criterion was according to the accuracy provided by DataMap. If the accuracy was more detailed than township (i.e., house number, POI, village, community, road, or intersection), the results of automatic geocoding by this route was regarded as reliable results. On the other hand, Python software (Version 3.9) was used to obtain the spatial coordinates of home addresses through the Tencent Maps Webservice API. The evaluation criterion was according to the reliability and the accuracy provided by Tencent Maps Webservice API. If the reliability was larger than or equal to 7 and the accuracy was larger than 3 (i.e., more detailed than township), the results of automatic geocoding was considered as reliable results. After automatic geocoding by the two methods simultaneously, the spatial coordinates of home addresses, which passed both the evaluation criteria, were regarded as the final reliable coordinates. To be noted, if the identified coordinates suggested that it was at other provinces instead of Beijing, this address was eliminated from the analysis below.

Second, for the reliable coordinates, we performed further verification by comparing the two obtained coordinated from Amap and Tencent Maps. The two maps both applied the GCJ-02 coordinate system, so they can be directly compared with each other. If the two coordinates were identical at the precision of three decimal places, the obtained coordinates were considered to be accurate addresses and directly used by the urban exposure assessment later. If the two coordinates were different at the precision of three decimal places, the distance between the two coordinates was further calculated. If the distance was shorter than 1 km, the coordinate was also considered as an accurate address. Otherwise, this home address would enter the step of manual verification.

Third, for unreliable results of automatic geocoding or the addresses which did not pass the test of distance, manual verification was conducted. Twenty volunteers were recruited and trained on the related methods. They should firstly pass an examination after the training, and can then engage in the manual verification. The volunteers should correct and supplement the home address information in the form of “City-District-Town-Village/Community/Road/Building” according to the current home address information in the Beijing Monitoring System. If the current address was too vague, the census registered home address was then referred to. The spatial coordinates were then obtained through Amap Webservice API. The information of accuracy, and whether to refer to census registered address were recorded by the volunteers. Five percent of the coordinates of home addresses obtained by each volunteer were randomly selected for examination by the first author of this study every three days, in order to guarantee the accuracy of manual verification. The working table during the manual verification is shown in **Figure S2**.

**Figure S2**. The working table in the step of manual verification. The headings of this table from left to right were identity number for hospital admission, unique personal identity number, registered home address, current home address, corrected home address, longitude, latitude, accuracy, whether to refer to registered home address during correction, and the change of accuracy due to correction.


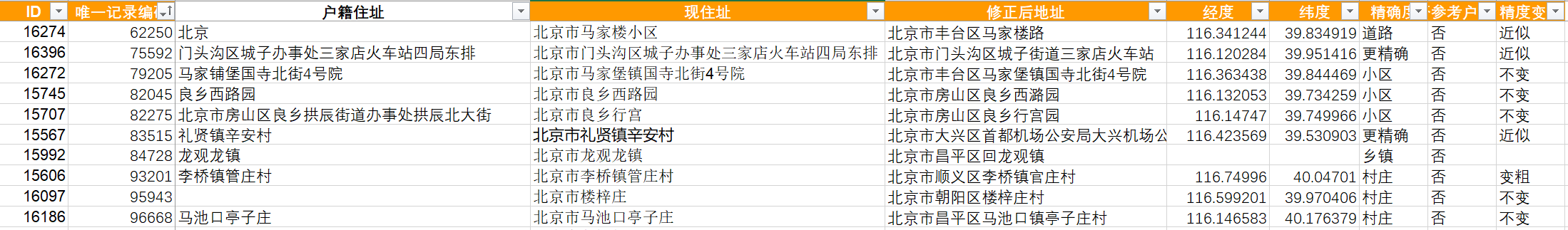


If the address obtained by manual verification can be more detailed than the township, it can also be considered as accurate addresses. Otherwise, the coordinate could only be categorized as inaccurate addresses, which was not included in the subsequent analysis.

**Section 2 Measurement of urban exposures**

This section introduces the methods of measuring each urban exposure in detail. Prior to the spatial data analysis, the region of Beijing was divided into 100 m 🞨 100 m grids in order to reduce the computational costs. Each grid was represented by its central point. All accurate home addresses obtained in Section 1 above were joined to these grids, and there were 61,368 grids in Beijing where there existed AMI patients. All the measurement of urban exposures below were based on the central points of grids instead of the exact spatial coordinates of each patient, as the 100-m resolution was assumed to be acceptable from the perspective of geocoding process. All aforementioned preprocessing was accomplished in ArcMap software (Version 10.2). All the subsequent spatial data analysis were conducted in QGIS software (Version 3.16.7) except additional statement. Information about data sources for each urban exposure was introduced in each subsection below, and was also summarized in **Table S1** at the end of this section.

**Figure S3**. The 100 m 🞨 100 m grids in Beijing.


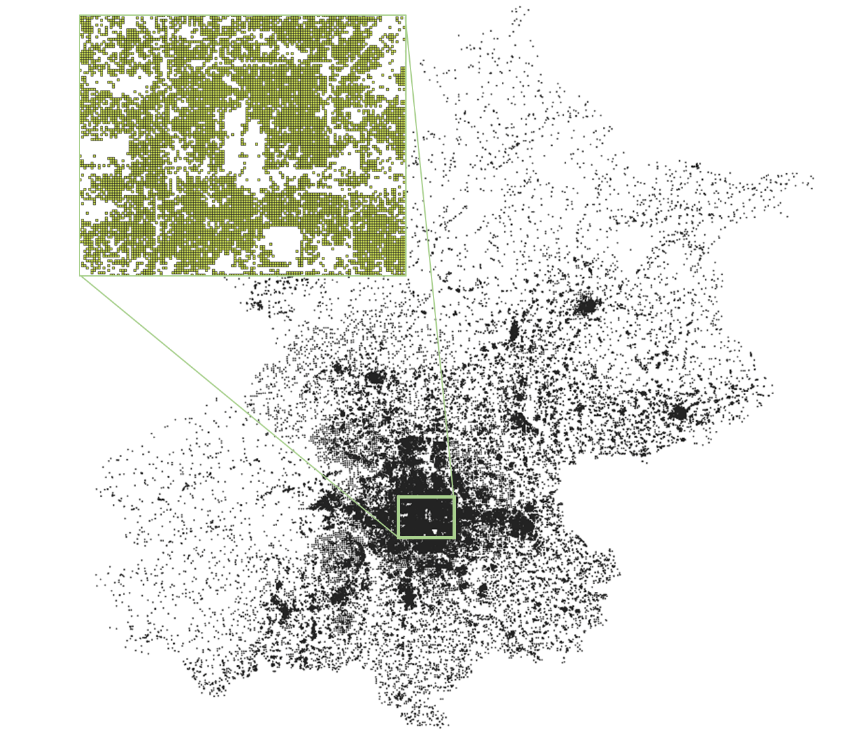


**2.1 Population density**

The population count data each year from 2013 to 2019 was acquired from WorldPop, and we selected the unconstrained 100-m resolution grid data adjusted to match UN population estimates ([WorldPop, 2018](#_ENREF_9)). First, we established 300-m, 500-m, and 1000-m buffers around the home addresses of the patients (i.e., the fishnet central points where the home address lies). Second, the grid data were transformed into the form of point features using the centroid of each grid, which were further joined to the buffers to obtain the total population counts in each buffer. Finally, the population counts were divided by the area of corresponding buffer, in order to obtain the population density.

**2.2 Building density and floor area ratio (FAR)**

The calculation of building density and FAR used the building data (polygon features) in 2014 and 2018 in Beijing, which were from Baidu Map. The data was only available within the 5^th^ Ring Road in Beijing. First, we established the 300-m, 500-m, and 1000-m buffers around the home addresses of the patients. Second, we calculated the area of each polygon to obtain the projected area of each building. Then the polygon features of buildings were transformed into the point features, which were joined to the buffers. For the building density, the projected area of buildings joined in each buffer was then summed up, and was further divided by the buffer area to obtain the building density. For the FAR, the floor area of buildings joined in each buffer was summed up. The data of floor area for each building in 2018 were not available, so they were estimated by the product of projected area and number of floors (The storey height was approximated as 3 meters). Then the FAR was calculated by dividing the total floor area in each buffer by the buffer area.

**2.3 Urban function density**

The urban function density was represented as the counts of POIs within the buffer. The data sources were the POI data from AMAP in 2011 and each year from 2014 to 2019. The point features of POIs were first joined to the buffers of patients’ home addresses. As the buffer area was the same among different home addresses, the counts of POIs in each buffer were regarded as the density of POIs, i.e. the urban function density.

**2.4 Land use diversity**

Land use data in 2014 were obtained from Beijing Institute of City Planning, which were in the form of polygon features. First, we established the 300-m, 500-m, and 1000-m buffers around the home addresses of the patients. Second, we classified the land use into eight types according to the Chinese national standard GB 50137-2011 ([Ministry of Housing and Urban-Rural Development of the People's Republic of China, 2011](#_ENREF_6)), including land for public service (A), commercial service (B), traffic (U), green space (G), public facilities (T), residence (R), industry and storage (M), and other uses. Then we intersected the land of different uses with the buffers, and merge the land of the same use within each buffer, to obtain the polygon features of land of different uses in each buffer. The area of each type of land in one buffer was calculated, and was divided by the total area of the corresponding type of land across Beijing to obtain the normalized area of this type of land in one buffer (denoted as *A*_i_). Next, the land use diversity was evaluated by a mixed land-use index, which was originated from the Shannon’s entropy index ([Long and Liu, 2013](#_ENREF_5)). The index in each buffer can be calculated by the equation below:

$M=-\sum_{i=1}^{8} p_{i}\ln p_{i}$ (1)

where

$p_{i}=\frac{A_{i}}{\sum_{i=1}^{8} A_{i}}$ (2)

**2.5 Urban function diversity**

The data sources were the POI data from AMAP for each year from 2015 to 2019. The calculation of urban function diversity was similar to that of land use diversity. The difference was that urban function diversity was based on different types of POIs instead of different types of land. We first classified all POIs into twelve types according to the classification of AMAP, including catering (code starts with 05), shopping (06), daily life service (07), sports and recreation (08), medical care (09), green space and parks (10), offices (1201, 1202, and 17), residence (1203), government (13), science, education and culture (14), traffic infrastructure (15 and 18), and others. Then, the counts of each type of POI in one buffer was divided by the total counts of the corresponding type of POI across Beijing to obtain the normalized count of this type of POI in one buffer (denoted as *N*_i_). Next, the urban function diversity in each buffer can be calculated by the equation below:

$M=-\sum_{i=1}^{12} p_{i}\ln p_{i}$ (3)

where

$p_{i}=\frac{N_{i}}{\sum_{i=1}^{12} N_{i}}$ (4)

**2.6 Road density**

The data of road were in the form of line features from AMAP in 2014, 2016, 2017, and 2019. As the raw data of roads may have repeated or over-detailed description of roads, each road was first simplified as single line feature, to avoid repeated calculation of road length. To be specific, 15-m buffer was established around each road, and all buffers were merged together and exported as files in the form of tiff. Then the ArcScan in the ArcMap (Version 10.2) was applied to extract the central line of each road, to obtain the simplified road network in the form of single lines. Second, we intersected the road network with the buffers around the patients’ home addresses. The total road length in each buffer can then be calculated. Finally, the road density was estimated by dividing the total road length in each buffer by the buffer area.

**2.7 Distance to main roads**

The data source was the road network (in the form of line features) from AMAP in 2016. The main roads were selected out according to the grade of roads, including the expressways, national roads, provincial roads, county roads, township roads, and urban first-class roads. Then we calculated the nearest distance from the home address to the main roads, which was considered as the distance to main roads.

**2.8 Physical disorder**

The physical disorder refers to the disturbance in residents’ lives and public spaces caused by observable or perceptible visual signs ([Chen et al., 2022](#_ENREF_2)). It can act as an external representation of urban decay and be associated with crimes and adverse outcomes. Chen et al. proposed an indicator system to quantify physical disorder for Chinese cities, including 5 types and 15 specific indicators. The 5 types of indicators for physical disorder are architecture (indicators: abandoned building, buildings with damaged facades, buildings with unkempt facades, graffiti/illegal advertisement, and illegal/temporary buildings), commerce (stores with poor signboards, stores with poor facades, and vacant and pending stores), road (broken roads, roads stacked with personal belongings, and garbage/litter on street), greening (messy and unmaintained greening), and other infrastructure (broken infrastructure, damaged public interface, and construction fence remnant) ([Chen et al., 2022](#_ENREF_2)).

The data source was Baidu Street View images within the 5^th^ Ring Road in Beijing in 2013, 2015, 2016, 2017, and 2019. In each street view image, the existence of each above indicator for physical disorder will add 1 score to the physical disorder score, so the score ranges from 0 to 15 and 15 scores suggest the worst physical disorder situation. The physical disorder scores within the 5^th^ Ring Road in Beijing have been assessed through deep learning for the street view images and published in other articles ([Chen et al., 2022](#_ENREF_2)). As there are four directions for each point of Baidu street view, we first calculated the average score of physical disorder among the four directions for each point. Based on the score of each point of street view, we calculated the average score of all points in the same buffer, to represent the physical disorder around the patients’ home addresses.

**2.9 Walkability**

The data source of walkability included the building data, road network data, and land use data within the 5^th^ Ring Road in Beijing in 2014. All these data have been described above. The calculation of walkability index followed the methods of Sallis et al ([Sallis et al., 2016](#_ENREF_7)). This index was based on the number of residential buildings, the density of intersections, and the land use diversity. The residential buildings were defined as those buildings whose central points were located in the residential land. Then the number of residential buildings can be counted in each buffer. The point features of the intersections can be directly obtained from the simplified road network mentioned in section 2.6. Then the density of intersections can be obtained by dividing the total counts of intersections in each buffer by the buffer area. The land use diversity has been explained in section 2.4. All the three values were further standardized by z-score normalization, respectively. The walkability index of each buffer was finally estimated by summing the three standardized indicators.

**2.10 Proportion of green space**

The data source for the proportion of green space has been described in section 2.4. We intersected the land of green space with the buffers. Then the area of green space in each buffer was divided by the buffer area to obtain the proportion of green space for each buffer.

**2.11 Green view index**

The data source of green view index was the Baidu Street View images within the 5^th^ Ring Road in Beijing in 2013, 2015, and 2017. All the images in these three years were taken in summer. The images in 2016 and 2019 were eliminated because they were taken in winter and leaves have all fallen. The green view index was estimated by the proportion of “green objects” in the street view image by the SegNet algorithm ([Li et al., 2015](#_ENREF_4)). The “green objects” included trees, grass, mountains, plants, flowers, and palms among the 150 objects in the SegNet algorithm. Similar to the physical disorder scores in Section 2.8, the green view index of each point of street view was first calculated by averaging the ratios of four directions. Then the green view index in each buffer was calculated by averaging the ratios of all points of street view in this buffer.

**2.12 Normalized difference vegetation index (NDVI)**

The data source was 30-m high-resolution Landsat 8 remote sensing images from the official website of United States Geological Survey (USGS). In order to guarantee the comparability and accuracy, only those images taken in summer with less than 5% of cloud coverage were included for the next analysis. Therefore, data in 2013, 2014, 2015, 2017, and 2019 in Beijing were available. The calculation of NDVI below was performed in ENVI software (Version 5.3). First, we conducted the radiometric calibration and atmospheric correction for the remote sensing images. Second, we can calculate the NDVI by the equation below:

$NDVI=\frac{NIR-R}{NIR+R}$ (5)

where *NIR* is the reflectivity in near infrared spectrum, and *R* is the reflectivity in red spectrum. The value NDVI should range from -1 to 1, so the abnormal values (accounting for only 0.5%) were forced to be -1 or 1. Using the zonal statistics in the QGIS, the average NDVI in each buffer can be finally obtained.

**2.13 Distance to or density of various POIs**

Lots of urban exposures in this study relied on the point features of POIs, including subway stations, bus stops, parks, tobacco and alcohol retailers, restaurants, fast food restaurants, dessert/drink/pastry shops, vegetable and fruit shops, sport venues, general hospitals, and pharmacies. The measurement method of the density of certain POI or the distance to the nearest certain POI was very similar, so it will be introduced in this part together. The data source was the POIs from AMAP from 2011 to 2019, and the details are shown in **Table S1**. The density of certain POI in one buffer can be calculated by the count number of this type of POI joined to this buffer. And the distance to the nearest certain type of POI can be directly conducted using QGIS software. To be noted, the distance was only measured for those rare POIs, including subway stations, parks, and general hospitals.

**2.14 Fine particulate matters (PM_2.5_)**

The data source was the 1-km resolution grid data of ambient PM_2.5_ concentration from the published article of Hammer et al ([Hammer et al., 2020](#_ENREF_3)). The exposure to PM_2.5_ can be directly estimated by the concentrations of the grid where the patient’s home address was located.

**Table S1**. Details about the data sources of urban exposures.

| Urban exposure | Data source | Time | Spatial resolution |
| --- | --- | --- | --- |
| Population density | WorldPop | 2013~2019 | 100-m grid data |
| Building density | Building data from Baidu Map | 2014, 2018 | Vector data (polygon) within the 5^th^ Ring Road in Beijing |
| Floor area ratio | Building data from Baidu Map | 2014, 2018 | Vector data (polygon) within the 5^th^ Ring Road in Beijing |
| Urban function density | POI data from AMAP | 2011, 2014~2019 | Vector data (point) |
| Land use diversity | Land use data from Beijing Institute of City Planning | 2014 | Vector data (polygon) |
| Urban function diversity | POI data from AMAP | 2011, 2014~2019 | Vector data (point) |
| Road density | Road data from AMAP | 2014, 2016, 2017, 2019 | Vector data (line) |
| Distance to main roads | Road data (major roads) from AMAP | 2016 | Vector data (line) |
| Physical disorder | Street view images from Baidu Map | 2013, 2015, 2016, 2017, 2019 | Streets with street view images within the 5^th^ Ring Road in Beijing |
| Walkability | Building data from Baidu Map;  Road data from AMAP;  Land use data from Beijing Institute of City Planning | 2014 | Vector data (polygon and line) within the 5^th^ Ring Road in Beijing |
| Proportion of green space | Land use data from Beijing Institute of City Planning | 2014 | Vector data (polygon) |
| Green view index | Street view images from Baidu Map | 2013, 2015, 2017 | Streets with street view images within the 5^th^ Ring Road in Beijing |
| NDVI | Remote sensing images from Landsat 8 | 2013, 2014, 2015, 2017, 2019 | 30-m grid data |
| Density of bus stops | POI data from AMAP (code: 1507XX) | 2014, 2016~2019 | Vector data (point) |
| Density of / Distance to subway stations | POI data from AMAP (code: 150500) | 2013~2019 | Vector data (point) |
| Density of / Distance to parks | POI data from AMAP (code: 1101XX) | 2011, 2014, 2016~2019 | Vector data (point) |
| Distance to large green space | Land use data from Beijing Institute of City Planning | 2014 | Vector data (polygon) |
| Density of tobacco and alcohol retailers | POI data from AMAP (code: 0602XX, 0604XX, 061210) | 2011, 2014~2019 | Vector data (point) |
| Density of fast food restaurants | POI data from AMAP (Western fried fast food restaurants in 0503XX) | 2011, 2014~2019 | Vector data (point) |
| Density of restaurants | POI data from AMAP (code: 050XXX) | 2011, 2014~2019 | Vector data (point) |
| Density of dessert / drink / pastry shop | POI data from AMAP (code: 050700, 050800, 050900) | 2011, 2014~2019 | Vector data (point) |
| Density of fruit and vegetable shop | POI data from AMAP (code: 0604XX, 060704, 060705) | 2011, 2014~2019 | Vector data (point) |
| Density of sport venue | POI data from AMAP (code: 0801XX, 0802XX) | 2011, 2014~2019 | Vector data (point) |
| Density of / Distance to general hospitals | POI data from AMAP (code: 0901XX) | 2011, 2014, 2016~2019 | Vector data (point) |
| Density of pharmacies | POI data from AMAP (code: 090601) | 2011, 2014, 2016~2019 | Vector data (point) |
| PM_2.5_ | Data products from articles in Environmental Science & Technology | 2013~2018 | 1-km grid data |

**Table S2**. Results of the urban exposure assessment and corresponding univariate analysis in the ExWAS for all the cohort across Beijing.

| Exposure | Baseline exposure | | | | Cumulative average exposure | | | |
| --- | --- | --- | --- | --- | --- | --- | --- | --- |
|  | Mean±SD or *N* (%) | IQR | HR (95% CI) | *p*-value | Mean±SD or *N* (%) | IQR | HR (95% CI) | *p*-value |
| Population density (person/km^2^) |  |  |  |  |  |  |  |  |
| 300-m buffer | 15244 ± 14403 | 19482 | 0.972 (0.945 ~ 1.000) | 0.054 | 15896 ± 14977 | 20347 | 0.908 (0.882 ~ 0.935) | < 0.001 |
| 500-m buffer | 15152 ± 13572 | 19832 | 0.970 (0.940 ~ 1.000) | 0.053 | 15808 ± 14114 | 20680 | 0.893 (0.865 ~ 0.922) | < 0.001 |
| 1000-m buffer | 14895 ± 12195 | 20608 | 0.952 (0.917 ~ 0.989) | 0.011 | 15544 ± 12681 | 21538 | 0.850 (0.818 ~ 0.883) | < 0.001 |
| Urban function density (number) |  |  |  |  |  |  |  |  |
| 300-m buffer | 281 ± 332 | 373 | 0.956 (0.932 ~ 0.979) | < 0.001 | 366 ± 356 | 430 | 0.588 (0.568 ~ 0.609) | < 0.001 |
| 500-m buffer | 750 ± 834 | 1055 | 0.939 (0.913 ~ 0.965) | < 0.001 | 979 ± 892 | 1225 | 0.535 (0.516 ~ 0.556) | < 0.001 |
| 1000-m buffer | 2774 ± 2929 | 4091 | 0.925 (0.897 ~ 0.954) | < 0.001 | 3621 ± 3108 | 4857 | 0.477 (0.458 ~ 0.497) | < 0.001 |
| Land use diversity |  |  |  |  |  |  |  |  |
| 300-m buffer | 1.01 ± 0.34 | 0.45 | 1.005 (0.981 ~ 1.030) | 0.698 | NA | NA | NA | NA |
| 500-m buffer | 1.23 ± 0.30 | 0.39 | 0.997 (0.973 ~ 1.020) | 0.774 | NA | NA | NA | NA |
| 1000-m buffer | 1.44 ± 0.26 | 0.33 | 0.998 (0.975 ~ 1.021) | 0.832 | NA | NA | NA | NA |
| Urban function diversity |  |  |  |  |  |  |  |  |
| 300-m buffer | 1.93 ± 0.58 | 0.46 | 0.963 (0.949 ~ 0.977) | < 0.001 | 2.02 ± 0.46 | 0.34 | 0.845 (0.834 ~ 0.856) | < 0.001 |
| 500-m buffer | 2.09 ± 0.49 | 0.31 | 0.968 (0.957 ~ 0.979) | < 0.001 | 2.15 ± 0.37 | 0.23 | 0.876 (0.867 ~ 0.885) | < 0.001 |
| 1000-m buffer | 2.22 ± 0.37 | 0.20 | 0.982 (0.973 ~ 0.991) | < 0.001 | 2.27 ± 0.28 | 0.13 | 0.919 (0.912 ~ 0.926) | < 0.001 |
| Road density (km/km^2^) |  |  |  |  |  |  |  |  |
| 300-m buffer | 10.7 ± 3.7 | 5.3 | 0.960 (0.934 ~ 0.987) | 0.004 | 10.8 ± 3.6 | 5.0 | 0.912 (0.887 ~ 0.939) | < 0.001 |
| 500-m buffer | 10.0 ± 3.5 | 5.2 | 0.935 (0.906 ~ 0.965) | < 0.001 | 10.1 ± 3.4 | 5.0 | 0.881 (0.854 ~ 0.910) | < 0.001 |
| 1000-m buffer | 9.2 ± 3.5 | 5.7 | 0.925 (0.890 ~ 0.960) | < 0.001 | 9.3 ± 3.4 | 5.5 | 0.863 (0.832 ~ 0.896) | < 0.001 |
| Distance to main roads (m) | 259.9 ± 296.8 | 240.2 | 0.994 (0.980 ~ 1.009) | 0.459 | NA | NA | NA | NA |
| Proportion of green space |  |  |  |  |  |  |  |  |
| 300-m buffer | 0.07 ± 0.12 | 0.08 | 1.014 (1.002 ~ 1.026) | 0.026 | NA | NA | NA | NA |
| 500-m buffer | 0.09 ± 0.13 | 0.10 | 1.019 (1.006 ~ 1.032) | 0.005 | NA | NA | NA | NA |
| 1000-m buffer | 0.12 ± 0.14 | 0.11 | 1.021 (1.008 ~ 1.035) | 0.002 | NA | NA | NA | NA |
| NDVI |  |  |  |  |  |  |  |  |
| 300-m buffer | 0.26 ± 0.10 | 0.12 | 1.008 (0.986 ~ 1.031) | 0.472 | 0.30 ± 0.10 | 0.10 | 0.901 (0.882 ~ 0.921) | < 0.001 |
| 500-m buffer | 0.28 ± 0.10 | 0.12 | 1.018 (0.995 ~ 1.040) | 0.125 | 0.29 ± 0.09 | 0.10 | 0.897 (0.878 ~ 0.916) | < 0.001 |
| 1000-m buffer | 0.30 ± 0.11 | 0.13 | 1.022 (0.999 ~ 1.046) | 0.064 | 0.32 ± 0.11 | 0.12 | 0.876 (0.855 ~ 0.898) | < 0.001 |
| Density of subway stations (number) |  |  |  |  |  |  |  |  |
| 300-m buffer | 0.1 ± 0.2 | 0 | 1.018 (0.942 ~ 1.101) | 0.646 | 0.06 ± 0.23 | 0 | 0.960 (0.888 ~ 1.038) | 0.308 |
| 500-m buffer | 0.2 ± 0.4 | 0 | 0.948 (0.904 ~ 0.995) | 0.03 | 0.19 ± 0.40 | 0 | 0.888 (0.846 ~ 0.932) | < 0.001 |
| 1000-m buffer | 0.8 ± 1.0 | 1 | 0.957 (0.937 ~ 0.977) | < 0.001 | 0.81 ± 0.96 | 1.17 | 0.893 (0.871 ~ 0.916) | < 0.001 |
| Distance to subway stations (m) | 5204.4 ± 9395.8 | 3849.7 | 1.030 (1.021 ~ 1.038) | < 0.001 | 4984.85 ± 9207.42 | 3323.36 | 1.036 (1.029 ~ 1.044) | < 0.001 |
| Density of bus stops (number) |  |  |  |  |  |  |  |  |
| 300-m buffer | 1.6 ± 1.7 | 2 | 0.993 (0.973 ~ 1.014) | 0.524 | 1.6 ± 1.6 | 1 | 0.985 (0.974 ~ 0.997) | 0.016 |
| 500-m buffer | 4.2 ± 3.5 | 4 | 0.986 (0.965 ~ 1.007) | 0.201 | 4.3 ± 3.2 | 4 | 0.954 (0.931 ~ 0.978) | < 0.001 |
| 1000-m buffer | 15.0 ± 10.3 | 12 | 0.985 (0.963 ~ 1.008) | 0.202 | 15.3 ± 9.8 | 12 | 0.952 (0.928 ~ 0.976) | < 0.001 |
| Density of parks (number) |  |  |  |  |  |  |  |  |
| 300-m buffer | 0.2 ± 0.5 | 0 | 0.975 (0.940 ~ 1.011) | 0.168 | 0.2 ± 0.5 | 0 | 0.910 (0.878 ~ 0.944) | < 0.001 |
| 500-m buffer | 0.6 ± 1.1 | 1 | 0.985 (0.968 ~ 1.003) | 0.104 | 0.6 ± 1.1 | 1 | 0.939 (0.922 ~ 0.957) | < 0.001 |
| 1000-m buffer | 2.4 ± 3.2 | 4 | 0.976 (0.952 ~ 1.002) | 0.066 | 2.7 ± 3.2 | 4 | 0.871 (0.847 ~ 0.897) | < 0.001 |
| Distance to parks (m) | 1160.4 ± 1541.5 | 768.5 | 1.010 (1.002 ~ 1.019) | 0.02 | 1040.9 ± 1249.3 | 672.6 | 1.062 (1.052 ~ 1.071) | < 0.001 |
| Distance to large green space (m) | 297.9 ± 264.2 | 287.3 | 0.990 (0.971 ~ 1.010) | 0.318 | NA | NA | NA | NA |
| Density of tobacco and alcohol retailers (number) |  |  |  |  |  |  |  |  |
| 300-m buffer | 8.2 ± 7.4 | 10 | 0.958 (0.932 ~ 0.985) | 0.003 | 9.8 ± 7.9 | 10 | 0.713 (0.693 ~ 0.734) | < 0.001 |
| 500-m buffer | 20.2 ± 16.8 | 24 | 0.944 (0.916 ~ 0.973) | < 0.001 | 24.3 ± 18.1 | 27 | 0.628 (0.607 ~ 0.650) | < 0.001 |
| 1000-m buffer | 69.6 ± 55.7 | 85 | 0.931 (0.901 ~ 0.963) | < 0.001 | 83.6 ± 60.1 | 95 | 0.579 (0.558 ~ 0.601) | < 0.001 |
| Density of fast food restaurants (number) |  |  |  |  |  |  |  |  |
| 300-m buffer | 0.4 ± 0.9 | 0 | 0.961 (0.942 ~ 0.980) | < 0.001 | 0.5 ± 1.0 | 1 | 0.892 (0.873 ~ 0.912) | < 0.001 |
| 500-m buffer | 1.1 ± 1.8 | 2 | 0.949 (0.927 ~ 0.971) | < 0.001 | 1.4 ± 2.0 | 2 | 0.848 (0.829 ~ 0.867) | < 0.001 |
| 1000-m buffer | 4.2 ± 4.7 | 6 | 0.934 (0.909 ~ 0.960) | < 0.001 | 5.0 ± 5.2 | 8 | 0.690 (0.665 ~ 0.716) | < 0.001 |
| Density of restaurants (number) |  |  |  |  |  |  |  |  |
| 300-m buffer | 29.6 ± 42.5 | 39 | 0.967 (0.946 ~ 0.988) | 0.003 | 37.9 ± 44.8 | 47 | 0.711 (0.691 ~ 0.731) | < 0.001 |
| 500-m buffer | 77.7 ± 98.5 | 106 | 0.963 (0.941 ~ 0.984) | < 0.001 | 99.5 ± 102.5 | 123 | 0.637 (0.617 ~ 0.658) | < 0.001 |
| 1000-m buffer | 279.3 ± 321.2 | 409 | 0.947 (0.921 ~ 0.973) | < 0.001 | 358.2 ± 327.8 | 458 | 0.556 (0.536 ~ 0.577) | < 0.001 |
| Density of dessert / drink / pastry shops (number) |  |  |  |  |  |  |  |  |
| 300-m buffer | 2.1 ± 4.3 | 2 | 0.988 (0.978 ~ 0.997) | 0.011 | 2.4 ± 4.2 | 3 | 0.926 (0.911 ~ 0.942) | < 0.001 |
| 500-m buffer | 5.6 ± 9.5 | 7 | 0.975 (0.961 ~ 0.990) | 0.001 | 6.5 ± 9.2 | 8 | 0.880 (0.862 ~ 0.899) | < 0.001 |
| 1000-m buffer | 20.5 ± 28.5 | 26 | 0.966 (0.948 ~ 0.984) | < 0.001 | 23.7 ± 26.1 | 32 | 0.796 (0.774 ~ 0.819) | < 0.001 |
| Density of fruit and vegetable shops (number) |  |  |  |  |  |  |  |  |
| 300-m buffer | 3.1 ± 3.6 | 4 | 0.951 (0.928 ~ 0.974) | < 0.001 | 4.0 ± 3.9 | 5 | 0.636 (0.616 ~ 0.657) | < 0.001 |
| 500-m buffer | 7.5 ± 7.5 | 9 | 0.941 (0.916 ~ 0.965) | < 0.001 | 9.7 ± 8.1 | 11 | 0.561 (0.543 ~ 0.581) | < 0.001 |
| 1000-m buffer | 25.2 ± 23.3 | 32 | 0.923 (0.895 ~ 0.951) | < 0.001 | 32.6 ± 25.4 | 39 | 0.471 (0.452 ~ 0.490) | < 0.001 |
| Density of sport venues (number) |  |  |  |  |  |  |  |  |
| 300-m buffer | 1.8 ± 2.9 | 2 | 0.985 (0.971 ~ 0.999) | 0.042 | 2.2 ± 3.2 | 3 | 0.830 (0.811 ~ 0.850) | < 0.001 |
| 500-m buffer | 4.9 ± 6.4 | 7 | 0.966 (0.943 ~ 0.989) | 0.004 | 6.0 ± 7.1 | 8 | 0.739 (0.717 ~ 0.761) | < 0.001 |
| 1000-m buffer | 18.9 ± 20.8 | 27 | 0.944 (0.917 ~ 0.972) | < 0.001 | 23.2 ± 22.9 | 32 | 0.618 (0.595 ~ 0.642) | < 0.001 |
| Density of general hospitals |  |  |  |  |  |  |  |  |
| 300-m buffer | 0.5 ± 0.5 | 1 | 0.993 (0.957 ~ 1.031) | 0.721 | 0.5 ± 0.5 | 1 | 1.029 (0.991 ~ 1.068) | 0.135 |
| 500-m buffer | 0.7 ± 0.4 | 1 | 1.001 (0.957 ~ 1.046) | 0.98 | 0.7 ± 0.4 | 1 | 1.032 (0.987 ~ 1.080) | 0.168 |
| 1000-m buffer | 0.9 ± 0.3 | 0 | 0.971 (0.915 ~ 1.031) | 0.337 | 0.9 ± 0.3 | 0 | 0.998 (0.940 ~ 1.059) | 0.94 |
| Distance to general hospital (m) | 514.9 ± 724.3 | 338.6 | 1.002 (0.993 ~ 1.011) | 0.699 | 534.6 ± 735 | 347 | 0.991 (0.981 ~ 1.000) | 0.062 |
| Density of pharmacies (number) |  |  |  |  |  |  |  |  |
| 300-m buffer | 1.3 ± 1.2 | 2 | 0.953 (0.922 ~ 0.984) | 0.004 | 1.4 ± 1.3 | 2 | 0.861 (0.834 ~ 0.888) | < 0.001 |
| 500-m buffer | 3.2 ± 2.4 | 4 | 0.955 (0.924 ~ 0.988) | 0.007 | 3.4 ± 2.5 | 4 | 0.819 (0.793 ~ 0.846) | < 0.001 |
| 1000-m buffer | 10.7 ± 7.4 | 12 | 0.938 (0.906 ~ 0.971) | < 0.001 | 11.6 ± 7.8 | 12 | 0.775 (0.750 ~ 0.801) | < 0.001 |
| PM_2.5_ (μg/m^3^) | 66.9 ± 17.6 | 32.2 | 1.087 (1.047 ~ 1.128) | < 0.001 | 57.3 ± 11.8 | 17 | 3.594 (3.502 ~ 3.689) | < 0.001 |

**Section 3** **Comparison of different variable selection algorithms**

The method of comparing different variable selection algorithms in the exposome approach followed the methods put forward by Agier et al. ^(^[Agier et al., 2016](#_ENREF_1)^)^ Here we introduced this method briefly and showed our comparison results for the data in this study.

**3.1 Methods**

There are six candidate algorithms in the Agier’s study, including ExWAS (Environment-wide association study), ExWAS-MLR (Environment-wide association study – Multiple linear regression), ENET (Elastic Net), sPLS (Sparse partial least squares regression), GUESS (Graphical unit evolutionary stochastic search), and DSA (Deletion-Substitution-Addition algorithm). As the R2GUESS package was too old and not applicable in R software currently, only other five algorithms were taken into account in this study.

**Generation of exposure data X.** We chose the AMI patients in the cohort who lived within the 5^th^ Ring Road and calculated the sample Pearson correlation matrix. The closest positive definite matrix Σ was then obtained based on the sample correlation matrix. Next, we can generate the exposure data which yield the multivariate normal distribution according to the aforementioned positive definite matrix Σ, i.e.,

$X\sim N\left(0,\Sigma\right)$ (6)

The sample size was selected as 12000, which was about 10% of the whole cohort.

**Generation of health outcome data Y.** The outcome Y was generated following

$Y=\sum_{i} \beta_{i}X_{i}+\varepsilon,\varepsilon\sim N\left(0,\sigma^{2}\right)$ (7)

where *X_i_* is the vector containing all values for the *i*th urban exposure and *ε* represents the residuals of the regression model. Regression coefficients *β_i_* were all set to 0 except for the *k* randomly selected exposures that were assumed to be related to the outcome for which *β_i_* = 1. These selected exposures were denoted as “true predictors”, and *k* can be 0, 1, 2, 3, 5, 10, 15, or 25.

**Main analysis (Scenario 1).** The correlation between the true predictors were unconstrained in the main analysis. The five variable selection algorithms were applied to fit the data, respectively. Then several indicators of statistical performance assessments can be obtained to evaluate these five algorithms. The random selection of true predictors and the model fit were conducted for 100 runs. The detailed statistical performance assessment was described below.

1. Sensitivity: the proportion of the true predictors selected by the algorithm in all the true predictors. (Larger is better)

$Sensitivity=N_{11}/N_{1+}$ (8)

where *N*_11_ is the number of true predictors selected by the algorithm, and *N*_1+_ is the number of all true predictors (i.e., *k*).

1. Alternative sensitivity: adjusted sensitivity which considers the selection of false exposures which was highly correlated with true predictors. (Larger is better)

$AltSens=\frac{1}{k}\sum_{i\in A} \max_{j\in B} \left|corr\left(X_{i},X_{j}\right)\right|$ (9)

where A is the set of true predictors, B is the set of exposures selected by the algorithm.

1. Specificity: the proportion of the false predictors not selected by the algorithm in all the false predictors. (Larger is better)

$Specificity=N_{22}/N_{2+}$ (10)

where *N*_22_ is the number of false predictors not selected by the algorithm, and *N*_2+_ is the number of all false predictors.

1. FDP (false discovery proportion): the proportion of the false predictors selected by the algorithm in all exposures selected by the algorithm. (Smaller is better)

$FDP=N_{21}/N_{+1}$ (11)

where *N*_21_ is the number of false predictors selected by the algorithm, and *N*_+1_ is the number of all exposures selected by the algorithm.

1. Alternative FDP: adjusted FDP which considers the selection of false exposures which was highly correlated with true predictors. (Smaller is better)

$AltFDP=1-\frac{1}{n_{B}}\sum_{j\in B} \max_{i\in A} \left|corr\left(X_{i},X_{j}\right)\right|$ (12)

1. *n*_B_/*k*: the ratio between the number of exposures selected by the algorithm and the number of true predictors (Closer to 1 is better)
2. MAE (mean absolute error) for all exposures (Smaller is better)

$MAE=\frac{1}{n_{total}}\sum_{i} \left|\beta_{i}-\hat{\beta}_{i}\right|$ (13)

where *β_i_* is the true regression coefficient, $\hat{\beta}_{i}$ is the fitted regression coefficient by the algorithm.

1. MAE for true predictors (Smaller is better)

$MAE\text{ }TP=\frac{1}{k}\sum_{i\in A} \left|\beta_{i}-\hat{\beta}_{i}\right|$ (14)

**Sensitivity analysis.** Several sensitivity analyses were conducted to test the stability of the performance for these algorithms. For scenario 2 and 3, the correlation between the true predictors was constrained to (0, 0.2) and (0.5, 1), respectively. For scenario 4 and 5, the coefficients in the correlation matrix Σ was multiplied by 0.5 (the coefficients on the main diagonal kept the value of 1) and 2 (the upper-bound of the coefficients were set as 1), respectively. For scenario 6, the exposure data was resampled from the true exposure data instead of being generated from a multivariate normal distribution, in order to explore the effect of skewed distribution on the algorithm performance. For scenario 7, the regression coefficients of the selected true predictors yield a uniform distribution ranging from 0.5 to 1.5. Each scenario was performed for 100 runs, respectively.

**3.2 Results**

**Table S3** and **Figure S4** show the results of statistical performance assessment of the main analysis (scenario 1) for each algorithm. We can conclude that the ranking of the five algorithms from the best to the poorest was DSA, ENET, sPLS, EWAS-MLR, and EWAS. In other words, the DSA algorithm has the best performance in the main analysis. The difference in the performance between DSA and ENET or sPLS is not quite significant, especially for small number of true predictors (less than 5). However, all the statistical performance except the sensitivity and alternative sensitivity of the ExWAS algorithm were much poorer than those of other algorithms. The FDP and alternative FDP of the ExWAS-MLR were also obviously poorer than the other models. It suggests that traditional hypothesis-based approach which focused on single exposures may be very unreliable.

The results of sensitivity analysis are shown in **Figure S5-S8**. From **Figure S5** for scenario 2 and 3, the larger the correlation is, the lower the sensitivity will be, and the higher the FDP and MAE will be. However, it should be noted that the correlation between true predictors does not influence the statistical performance of the algorithms very much, especially for ENET, sPLS, and DSA. Nevertheless, the correlation matrix can exert significant impacts on the performance of these algorithms, as is shown in **Figure S6** for scenario 4 and 5. The larger the coefficients in the correlation matrix are, the poorer the performance of all algorithms will be. The sensitivity and alternative sensitivity of ExWAS and sPLS keeps relatively stable. The FDPs of all algorithms were all poor in scenario 5, but the alternative FDP of each algorithm was very robust. In particular, the AltFDP of ENET has the best performance. From the perspective of specificity, ExWAS-MLR and ENET have relatively stable and better performance. For the MAE, ENET and sPLS are more stable when the coefficients of correlation matrix increase. From **Figure S7** for scenario 6, we can infer that the skewed distribution has little effect on the statistical performance of these algorithms. Finally, **Figure S8** for scenario 7 shows that the variable regression coefficient also has little effect on the algorithm performance, except that the FDP and specificity of ENET can become better.

In conclusion, the DSA algorithm has the best performance in the main analysis, but the ENET algorithm has the best performance in the sensitivity analysis. Besides, in the main analysis, the performance of ENET is very close to that of DSA. Additionally, we considered that for current packages in R, it’s easy to combine the DSA algorithm with linear regression and generalized linear models but difficult with Cox proportional hazard model. Therefore, in the main text in this study, we combined the Cox proportional hazard model with the ENET algorithm to realize the variable selection.

**Table S3**. Mean [min, max] of the statistical performance assessment in the main analysis (scenario 1) for five algorithms for 100 runs.

|  | Sensitivity | AltSens | FDP | AltFDP | Specificity | *n*_B_/*k* | MAE | MAE for TP |
| --- | --- | --- | --- | --- | --- | --- | --- | --- |
| EWAS | 0.99 [0.98, 1.00] | 1.00 [0.99, 1.00] | 0.89 [0.69, 0.97] | 0.54 [0.29, 0.73] | 0.27 [0.04, 1.00] | 18.74 [3.22, 46.92] | 0.91 [0.07, 2.72] | 0.92 [0.04, 2.62] |
| EWAS-MLR | 0.98 [0.96, 1.00] | 0.99 [0.98, 1.00] | 0.36 [0.13, 0.57] | 0.23 [0.05, 0.45] | 0.96 [0.93, 1.00] | 1.88 [1.14, 3.21] | 0.09 [0.00, 0.13] | 0.12 [0.09, 0.15] |
| ENET | 1.00 [0.99, 1.00] | 1.00 [0.99, 1.00] | 0.16 [0.00, 0.29] | 0.04 [0.00, 0.08] | 0.96 [0.86, 1.00] | 1.23 [1.00, 1.42] | 0.02 [0.00, 0.06] | 0.34 [0.17, 0.60] |
| sPLS | 0.99 [0.98, 1.00] | 1.00 [0.99, 1.00] | 0.32 [0.10, 0.55] | 0.12 [0.06, 0.17] | 0.88 [0.54, 1.00] | 1.72 [1.19, 2.31] | 0.04 [0.00, 0.14] | 0.13 [0.05, 0.26] |
| DSA | 1.00 [0.99, 1.00] | 1.00 [1.00, 1.00] | 0.05 [0.01, 0.11] | 0.03 [0.01, 0.09] | 1.00 [0.99, 1.00] | 1.10 [1.01, 1.34] | 0.01 [0.00, 0.02] | 0.06 [0.04, 0.08] |

**Figure S4**. Mean of the statistical performance assessment in the main analysis (scenario 1) for five algorithms for 100 runs. Notes: the dot *k*=0 was only available in the specificity and MAE.


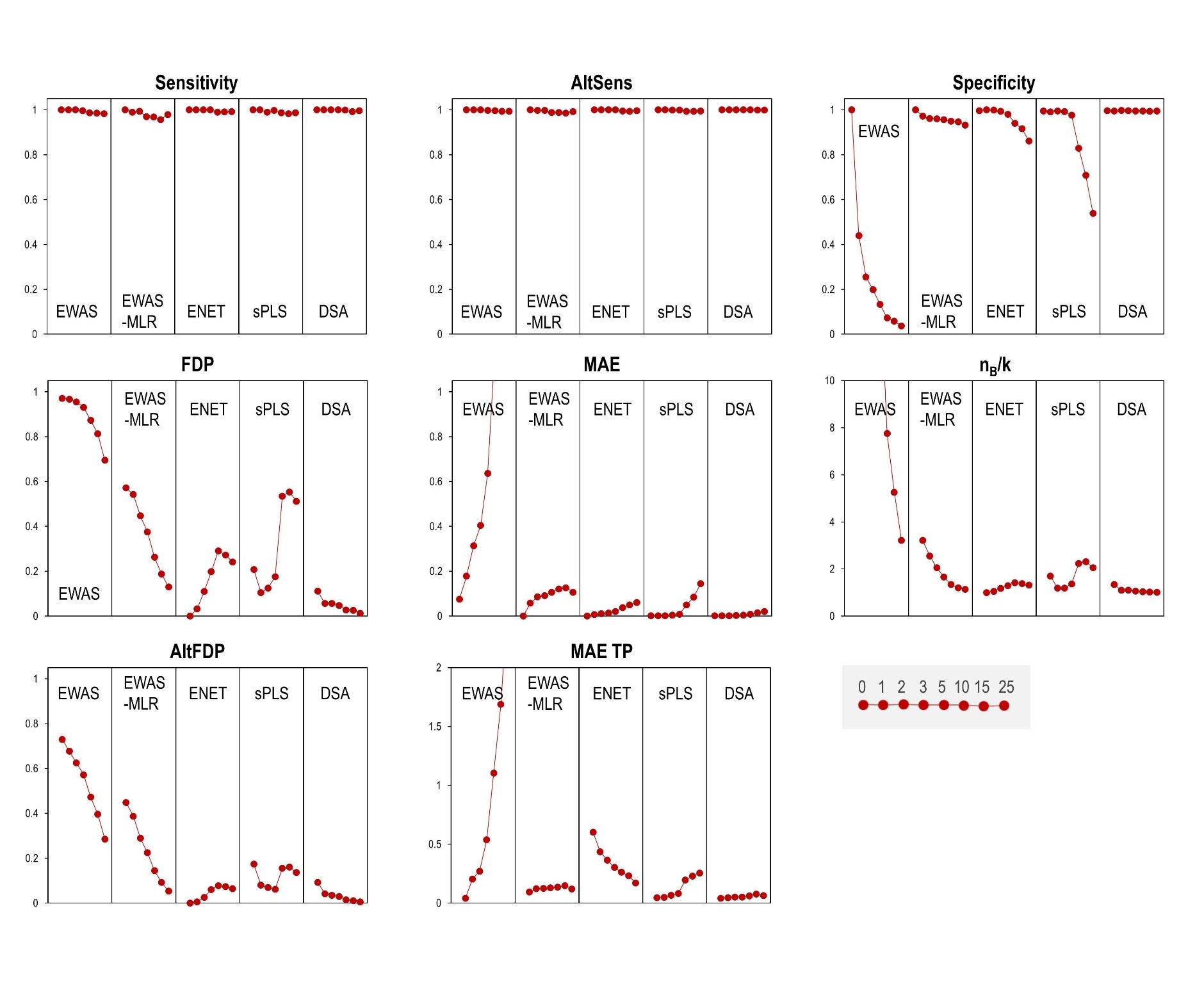


**Figure S5**. Mean of the statistical performance assessment in scenario 2 and 3 for five algorithms for 100 runs. Notes: the dot *k*=0 was only available in the specificity and MAE.


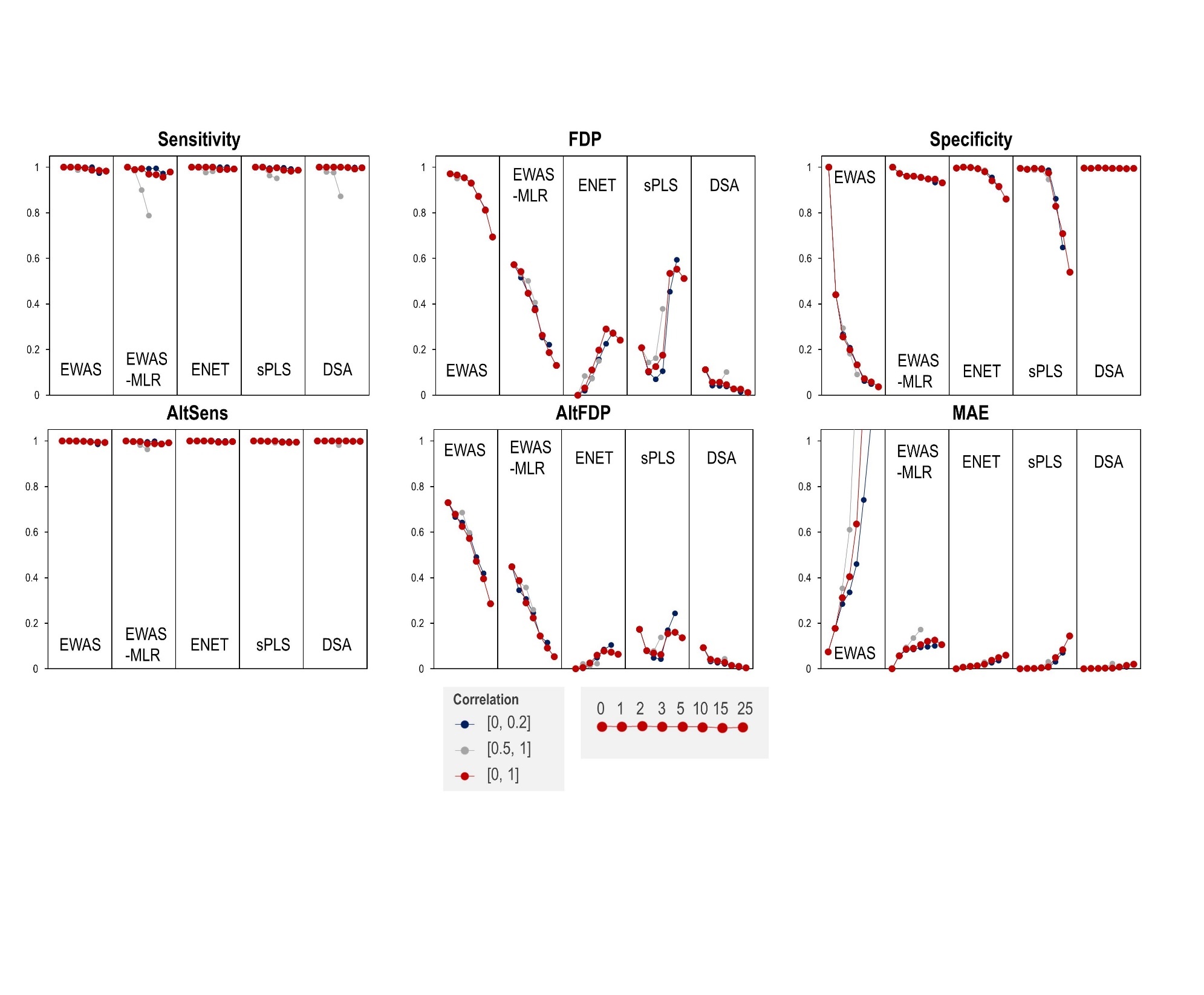


**Figure S6**. Mean of the statistical performance assessment in scenario 4 and 5 for five algorithms for 100 runs. Notes: the dot *k*=0 was only available in the specificity and MAE.


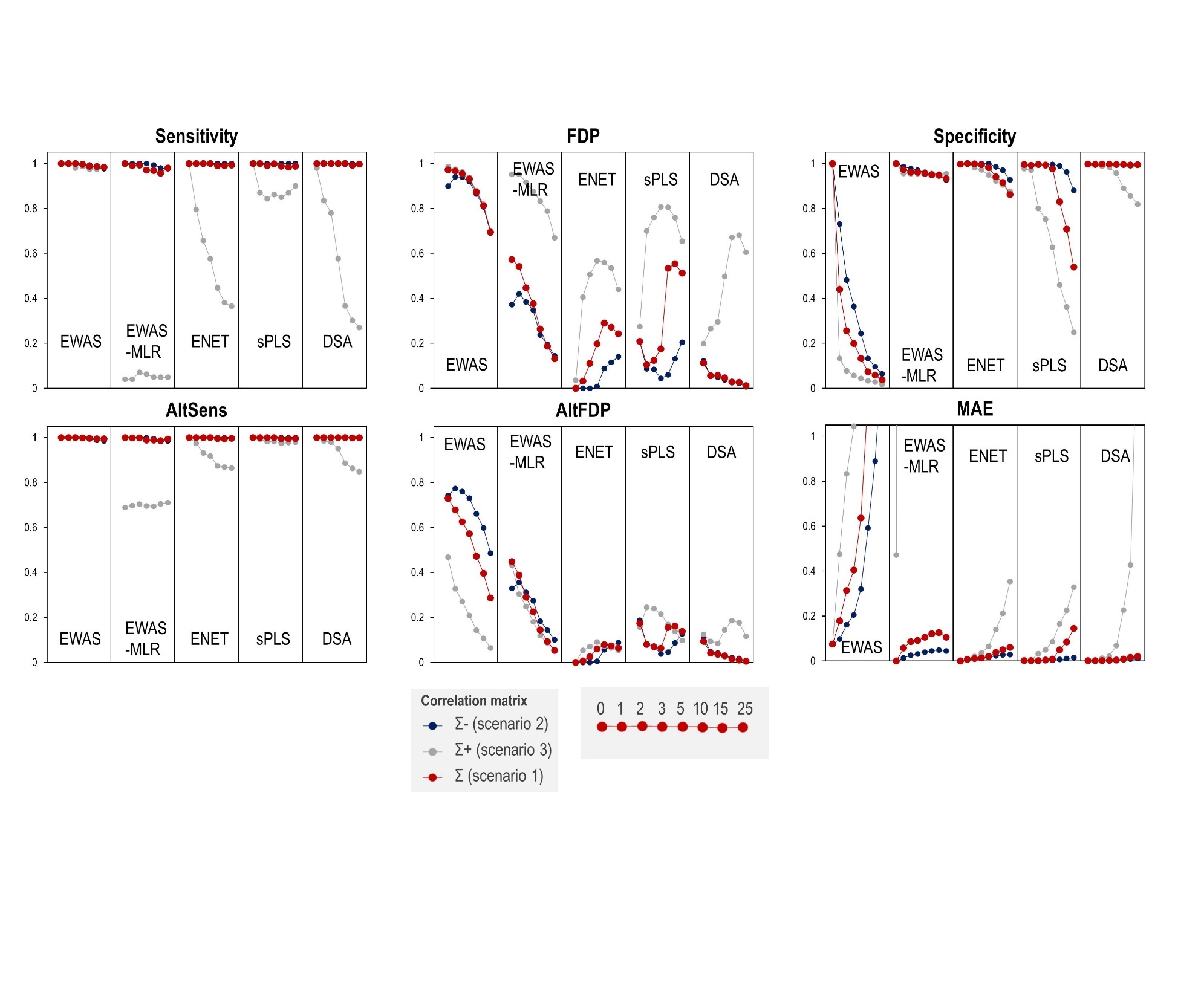


**Figure S7**. Mean of the statistical performance assessment in scenario 6 for five algorithms for 100 runs. Notes: the dot *k*=0 was only available in the specificity and MAE.


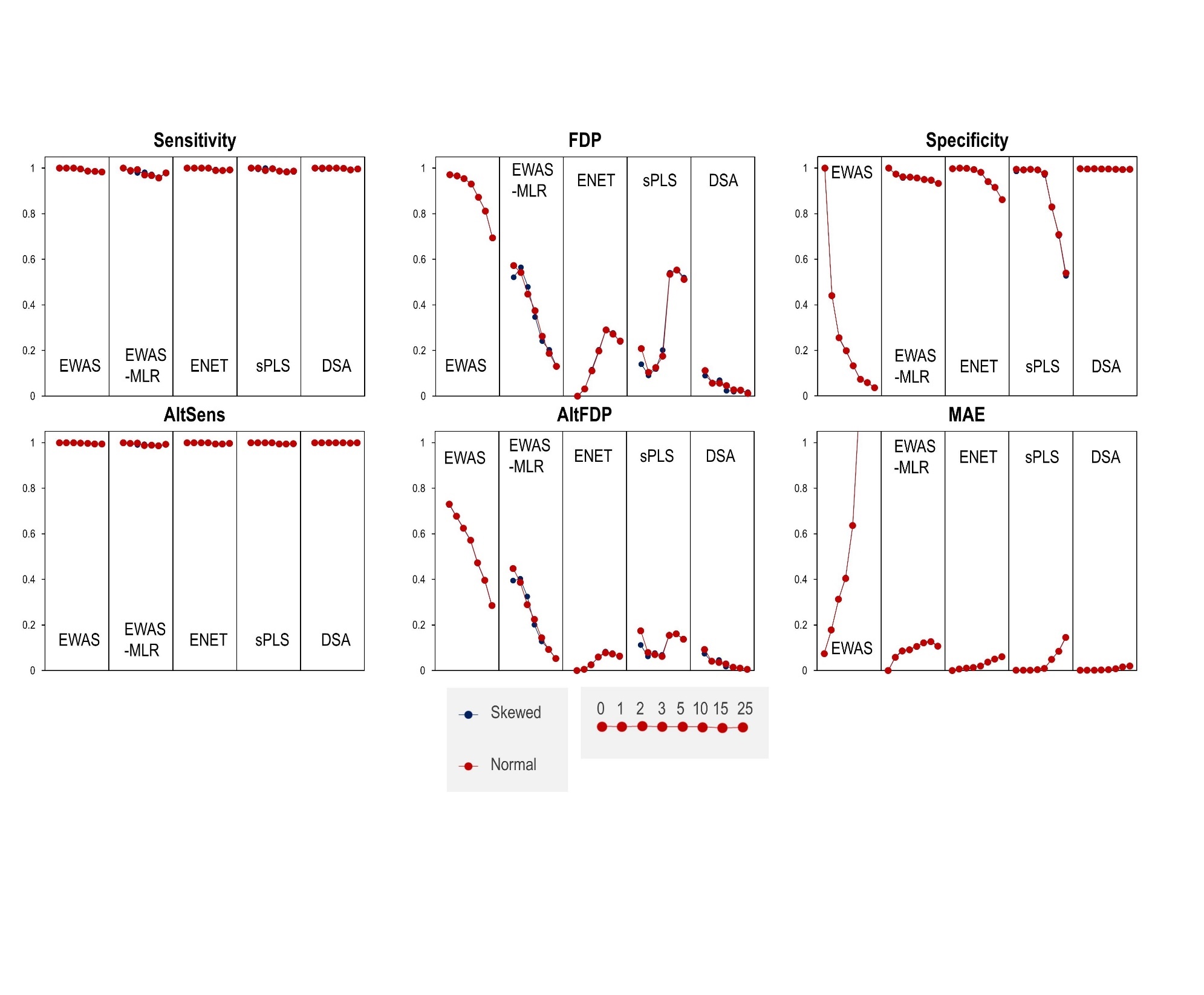


**Figure S8**. Mean of the statistical performance assessment in scenario 7 for five algorithms for 100 runs. Notes: the dot *k*=0 was only available in the specificity and MAE.


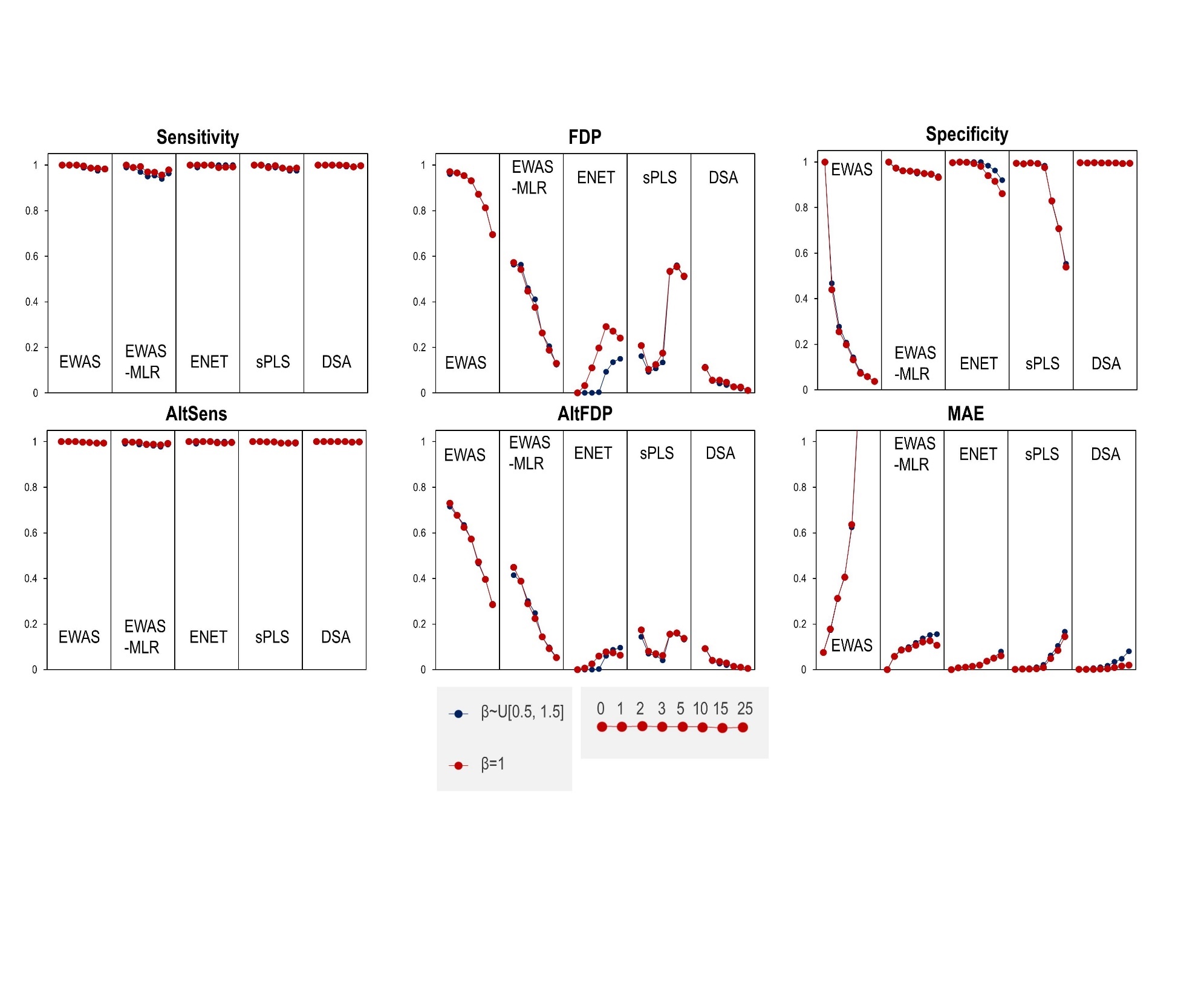


**Section 4** **Diagnostics and remedies of the Cox proportional hazard model**

This section first introduced the detailed description about the Cox proportional hazard model and the extended Cox model used in this study. In the survival analysis, one important concept is the survivor function *S*(*t*), which is defined as the probability of the survivor time longer than *t*, i.e.,

$S\left(t\right)=P\left(T>t\right)=1-F\left(t\right)=\int_{t}^{\infty} f\left(t\right)dt$ (15)

where *S*(*t*) is the survivor function, *F*(*t*) is the cumulative distribution function of survivor time, and *f*(*t*) is the probability density function of survivor time.

Then the hazard function can be defined as,

$h\left(t\right)=\lim_{h\to0^{+}} \frac{P\left(t\leq T<t+h\left|T\geq t\right.\right)}{h}=\frac{f\left(t\right)}{S\left(t\right)}=-\frac{d\ln S\left(t\right)}{dt}$ (16)

where *h*(*t*) is the hazard function. It can be also seen that the hazard function is proportional to the first derivative of the natural logarithm of *S*(*t*).

The Cox proportional hazard model is to model the association between the hazard function and multiple explanatory variables, to assess the effect of these factors on the incidence risk of the outcome. The formula of Cox proportional hazard model can be written as,

$h\left(t\right)=h_{0}\left(t\right)\exp\left(\sum_{i} \beta_{i}X_{i}\right)$ (17)

where *h*_0_(*t*) is the baseline hazard when all *X_i_* equal 0. The proportional hazard (PH) assumption refers to that the ratio of hazard function and the baseline hazard is constant over time. If the explanatory variable *X_k_* does not meet the PH assumption, an extended Cox model will be applied as below,

$h\left(t\right)=h_{0}\left(t\right)\exp\left(\sum_{i} \beta_{i}X_{i}+\gamma_{k}\ln\left(t\right)X_{k}\right)$ (18)

where *X_k_* is the variable not meeting the PH assumption, *γ_k_* is the regression coefficient of the interaction term between *X_k_* and the natural logarithm of time. Then, the hazard ratio (HR) will vary with time, i.e.,

$HR_{k}\left(t\right)=\exp\left(\beta_{k}+\gamma_{k}\ln\left(t\right)\right)$ (19)

If several explanatory variables simultaneously do not meet the PH assumption, the extended Cox model can be given very similar to equation (18) by including interaction terms with each of those variables.

**Table S4**. Results of Schoenfeld’s residuals tests for selected urban exposure variables for all the cohort in Beijing, China.

| Variable | All recurrent AMI | Fatal recurrent AMI | Nonfatal recurrent AMI |
| --- | --- | --- | --- |
| Baseline exposure | | | |
| Urban function diversity (500-m buffer) | *p* = 0.669 | *p* = 0.147 | *p* = 0.943 |
| Road density (500-m buffer) | *p* = 0.109 | *p* = 0.066 | *p* = 0.327 |
| Proportion of green space (1000-m buffer) | *p* = 0.345 | *p* = 0.057 | *p* = 0.845 |
| Distance to subway stations | ***p* < 0.001** | ***p* < 0.001** | ***p* < 0.001** |
| Density of fast food restaurants (300-m buffer) | *p* = 0.637 | *p* = 0.115 | *p* = 0.860 |
| Density of fruit and vegetable shops (300-m buffer) | *p* = 0.106 | *p* = 0.427 | *p* = 0.186 |
| Density of pharmacies (300-m buffer) | *p* = 0.736 | *p* = 0.560 | *p* = 0.786 |
| PM_2.5_ | ***p* < 0.001** | ***p* < 0.001** | ***p* = 0.004** |
| Cumulative average exposure | | | |
| Urban function diversity (500-m buffer) | ***p* = 0.032** | *p* = 0.104 | *p* = 0.225 |
| Distance to subway stations | ***p* < 0.001** | ***p* < 0.001** | ***p* < 0.001** |
| Distance to parks | *p* = 0.687 | ***p* = 0.033** | *p* = 0.881 |
| Density of fruit and vegetable shops (1000-m buffer) | ***p* < 0.001** | *p* = 0.520 | *p* = 0.123 |
| PM_2.5_ | ***p* < 0.001** | *p* = 0.053 | *p* = 0.454 |

**Table S5**. Results of Schoenfeld’s residuals tests for selected urban exposure variables for the sub-cohort within the 5^th^ Ring Road in Beijing, China.

| Variable | All recurrent AMI | Fatal recurrent AMI | Nonfatal recurrent AMI |
| --- | --- | --- | --- |
| Baseline exposure | | | |
| Distance to parks | ***p* = 0.035** | *p* = 0.067 | *p* = 0.127 |
| Density of restaurants (1000-m buffer) | *p* = 0.184 | ***p* = 0.010** | *p* = 0.288 |
| Density of fast food restaurants (1000-m buffer) | *p* = 0.263 | ***p* = 0.018** | *p* = 0.546 |
| Density of fruit and vegetable shops (1000-m buffer) | *p* = 0.980 | *p* = 0.127 | *p* = 0.621 |
| PM_2.5_ | *p* = 0.122 | *p* = 0.081 | *p* = 0.176 |
| Cumulative average exposure | | | |
| PM_2.5_ | ***p* < 0.001** | *p* = 0.811 | *p* = 0.924 |

**Section 5** **Correlation among different urban exposure variables**

**Figure S9**. The spearman correlation matrix for (*a*) baseline exposure level and (*b*) cumulative average exposure level of all urban exposure variables.


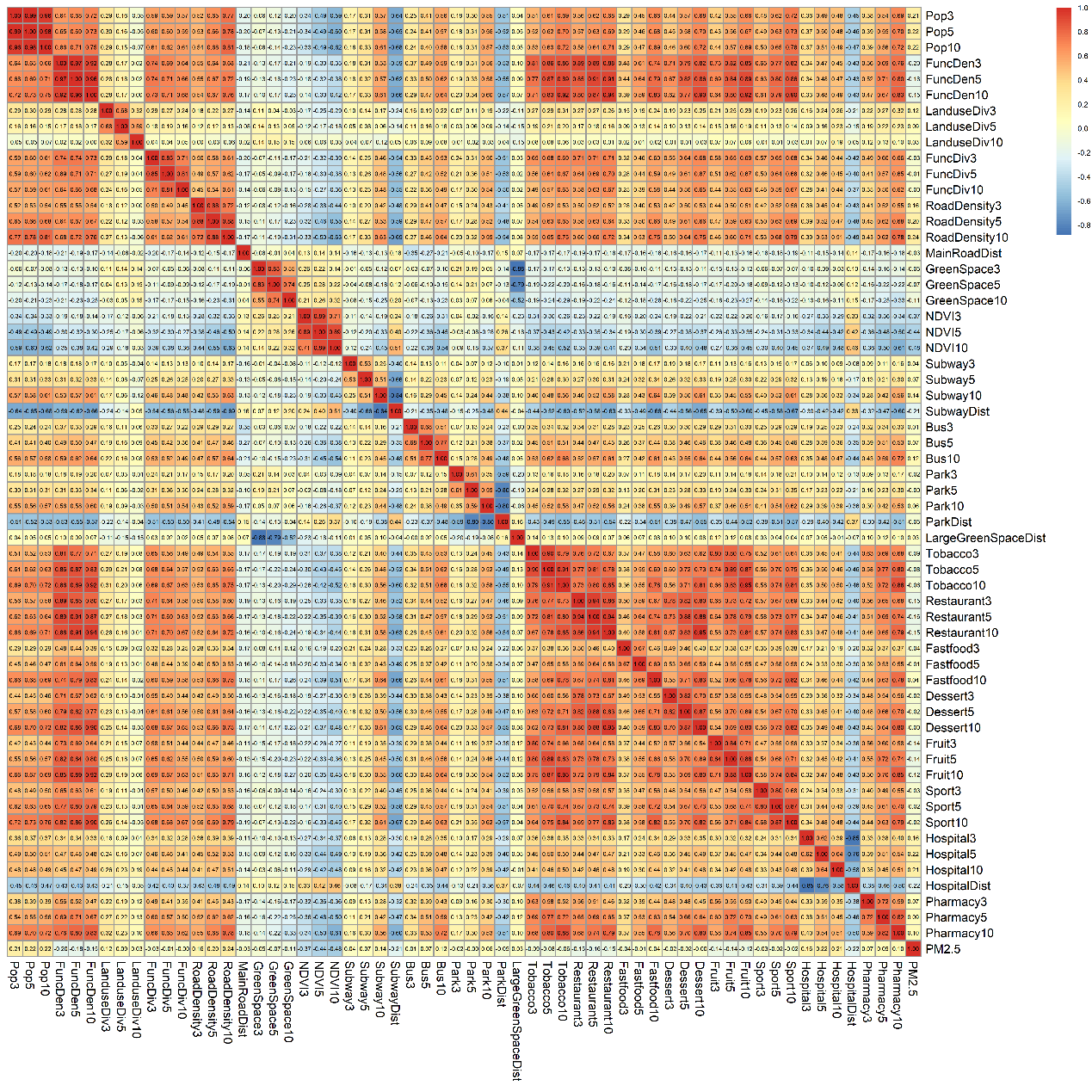


(*a*) Baseline exposure


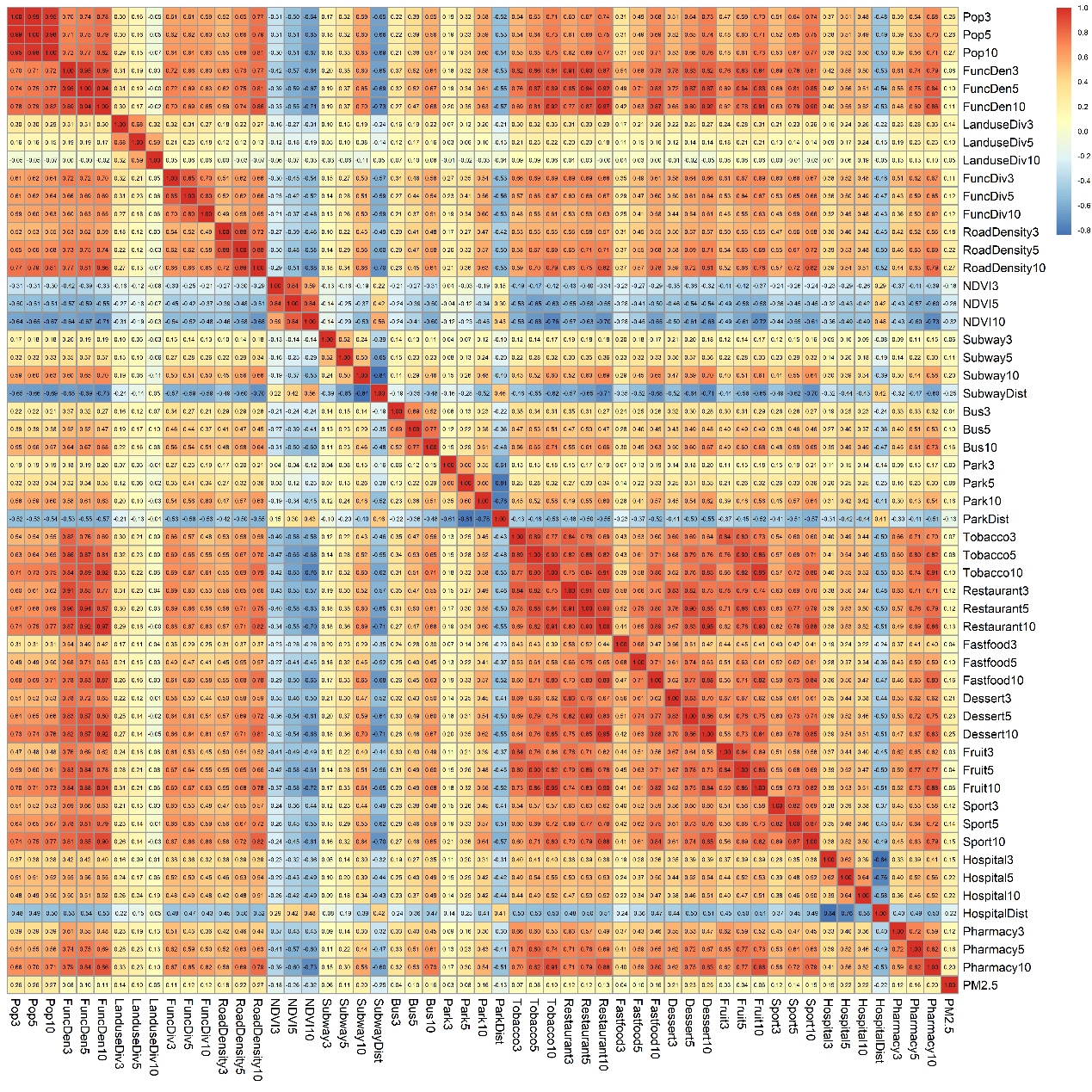


(*b*) Cumulative average exposure

Note: The “3”, “5”, and “10” in the labels represent 300-m, 500-m, and 1000-m buffer. Pop = population density, FuncDen = urban function density, FuncDiv = urban function diversity, RoadDensity = road density, MainRoadDist = distance to main roads, GreenSpace = proportion of green space, NDVI = Normalized Difference Vegetation Index, Subway = existence of subway stations, SubwayDist = distance to the nearest subway station, Bus = density of bus stations, ParkDist = distance to parks, LargeGreenSpaceDist = distance to large green space, Tobacco = density of tobacco and alcohol shops, Restaurant = density of restaurants, Fastfood = density of fast food restaurants, Dessert = density of dessert, drink and pastry shops, Fruit = density of fruit and vegetable shops, Sport = density of sport venues, Hospital = density of general hospitals, HospitalDist = distance to the nearest general hospital, Pharmacy = density of pharmacies, PM2.5 = PM_2.5_ concentration.

**Section 6** **Results for AMI patients living in the 5^th^ Ring Road in Beijing**

**Table S6**. Adjusted associations between the baseline exposure level of selected urban exposures and recurrent AMI events for patients living in the 5^th^ Ring Road in Beijing through the Cox model.

| Variable | Buffer | IQR | All recurrent AMI |  | Fatal recurrent AMI |  | Nonfatal recurrent AMI | |
| --- | --- | --- | --- | --- | --- | --- | --- | --- |
|  |  |  | HR (95% CI) *^a^* | *p* value | HR (95% CI) *^a^* | *p* value | HR (95% CI) *^a^* | *p* value |
| Baseline exposure |  |  |  |  |  |  |  |  |
| Distance to parks (km) | —— | 0.428 | **1.036 (1.003 to 1.070)** *^c^* | 0.030 | 0.970 (0.916 to 1.028) | 0.303 | **1.050 (1.013 to 1.090)** *^b^* | 0.009 |
| 1 week (7 days) |  |  | 1.075 (0.994 to 1.163) | 0.072 |  |  |  |  |
| 1 month (30 days) |  |  | 1.054 (0.965 to 1.151) | 0.244 |  |  |  |  |
| 3 months (90 days) |  |  | 1.038 (0.941 to 1.145) | 0.453 |  |  |  |  |
| 6 months (180 days) |  |  | 1.028 (0.926 to 1.142) | 0.600 |  |  |  |  |
| 1 year (365 days) |  |  | 1.019 (0.911 to 1.139) | 0.748 |  |  |  |  |
| 2 years (730 days) |  |  | 1.009 (0.895 to 1.137) | 0.884 |  |  |  |  |
| 3 years (1095 days) |  |  | 1.003 (0.886 to 1.136) | 0.958 |  |  |  |  |
| 5 years (1825 days) |  |  | 0.996 (0.875 to 1.135) | 0.957 |  |  |  |  |
| Density of restaurants (number) | 1000 m | 479 | 0.985 (0.934 to 1.039) | 0.586 | 1.068 (0.976 to 1.169) *^c^* | 0.152 | 0.941 (0.884 to 1.002) | 0.059 |
| 1 week (7 days) |  |  |  |  | 1.175 (0.941 to 1.469) | 0.155 |  |  |
| 1 month (30 days) |  |  |  |  | 1.118 (0.871 to 1.434) | 0.381 |  |  |
| 3 months (90 days) |  |  |  |  | 1.076 (0.817 to 1.419) | 0.601 |  |  |
| 6 months (180 days) |  |  |  |  | 1.051 (0.783 to 1.412) | 0.741 |  |  |
| 1 year (365 days) |  |  |  |  | 1.026 (0.748 to 1.407) | 0.874 |  |  |
| 2 years (730 days) |  |  |  |  | 1.002 (0.715 to 1.403) | 0.992 |  |  |
| 3 years (1095 days) |  |  |  |  | 0.988 (0.696 to 1.402) | 0.945 |  |  |
| 5 years (1825 days) |  |  |  |  | 0.971 (0.673 to 1.400) | 0.873 |  |  |
| Density of fast food restaurants (number) | 1000 m | 6 | 0.987 (0.945 to 1.031) | 0.554 | 0.943 (0.874 to 1.018) *^c^* | 0.136 | 1.006 (0.956 to 1.058) | 0.817 |
| 1 week (7 days) |  |  |  |  | 0.973 (0.790 to 1.198) | 0.794 |  |  |
| 1 month (30 days) |  |  |  |  | 0.958 (0.759 to 1.209) | 0.719 |  |  |
| 3 months (90 days) |  |  |  |  | 0.948 (0.733 to 1.225) | 0.681 |  |  |
| 6 months (180 days) |  |  |  |  | 0.941 (0.715 to 1.238) | 0.663 |  |  |
| 1 year (365 days) |  |  |  |  | 0.934 (0.697 to 1.252) | 0.648 |  |  |
| 2 years (730 days) |  |  |  |  | 0.927 (0.678 to 1.268) | 0.637 |  |  |
| 3 years (1095 days) |  |  |  |  | 0.924 (0.668 to 1.278) | 0.631 |  |  |
| 5 years (1825 days) |  |  |  |  | 0.919 (0.654 to 1.291) | 0.625 |  |  |
| Density of fruit and vegetable shops (number) | 1000 m | 31 | 0.967 (0.916 to 1.022) | 0.233 | 0.969 (0.879 to 1.069) | 0.528 | 0.970 (0.910 to 1.032) | 0.334 |
| PM_2.5_ (μg/m^3^) | —— | 33.600 | 1.036 (0.960 to 1.119) | 0.366 | **1.238 (1.076 to 1.423)** *^b^* | 0.003 | 1.007 (0.922 to 1.100) | 0.872 |

*^a^* Coefficients are provided for an IQR increase in the given variable of urban exposome, adjusted by age, sex, marital status, average education years, average annual household income, and the history of complications (hyperlipidemia, diabetes, stroke, heart failure, atrial fibrillation, COPD, and renal dysfunction).

*^b^* *p* < 0.05

*^c^* Coefficients are provided by the Cox proportional hazard model (i.e., constant HRs) instead of the extended model with time-varying coefficients.

**Table S7**. Adjusted associations between the cumulative average exposure level of selected urban exposures and recurrent AMI events for patients living in the 5^th^ Ring Road in Beijing through the Cox model.

| Variable | Buffer | IQR | All recurrent AMI |  | Fatal recurrent AMI |  | Nonfatal recurrent AMI | |
| --- | --- | --- | --- | --- | --- | --- | --- | --- |
|  |  |  | HR (95% CI) *^a^* | *p* value | HR (95% CI) *^a^* | *p* value | HR (95% CI) *^a^* | *p* value |
| PM_2.5_ (μg/m^3^) | —— | 18.383 | **5.704 (5.456 to 5.962)** *^c^* | < 0.001 | **1.100 (1.008 to 1.201)** *^b^* | 0.033 | **1.061 (1.007 to 1.119)** *^b^* | 0.026 |
| 1 week (7 days) |  |  | **3.616 (3.269 to 4.000)** *^b^* | < 0.001 |  |  |  |  |
| 1 month (30 days) |  |  | **5.330 (4.737 to 5.997)** *^b^* | < 0.001 |  |  |  |  |
| 3 months (90 days) |  |  | **7.143 (6.245 to 8.170)** *^b^* | < 0.001 |  |  |  |  |
| 6 months (180 days) |  |  | **8.592 (7.426 to 9.941)** *^b^* | < 0.001 |  |  |  |  |
| 1 year (365 days) |  |  | **10.373 (8.856 to 12.151)** *^b^* | < 0.001 |  |  |  |  |
| 2 years (730 days) |  |  | **12.478 (10.519 to 14.802)** *^b^* | < 0.001 |  |  |  |  |
| 3 years (1095 days) |  |  | **13.902 (11.632 to 16.616)** *^b^* | < 0.001 |  |  |  |  |
| 5 years (1825 days) |  |  | **15.930 (13.200 to 19.224)** *^b^* | < 0.001 |  |  |  |  |

*^a^* Coefficients are provided for an IQR increase in the given variable of urban exposome, adjusted by age, sex, marital status, average education years, average annual household income, and the history of complications (hyperlipidemia, diabetes, stroke, heart failure, atrial fibrillation, COPD, and renal dysfunction).

*^b^* *p* < 0.05

*^c^* Coefficients are provided by the Cox proportional hazard model (i.e., constant HRs) instead of the extended model with time-varying coefficients.

**References**

Agier, L., et al., 2016. A Systematic Comparison of Linear Regression-Based Statistical Methods to Assess Exposome-Health Associations. Environ Health Persp. 124**,** 1848-1856.

Chen, J., et al., 2022. Measuring physical disorder in urban street spaces: A large-scale analysis using street view images and deep learning. Ann Am Assoc Geogr. 113**,** 469-487.

Hammer, M. S., et al., 2020. Global Estimates and Long-Term Trends of Fine Particulate Matter Concentrations (1998-2018). Environ Sci Technol. 54**,** 7879-7890.

Li, X., et al., 2015. Assessing street-level urban greenery using Google Street View and a modified green view index. Urban Forestry & Urban Greening. 14**,** 675-685.

Long, Y., Liu, X., 2013. Featured Graphic. How Mixed is Beijing, China? A Visual Exploration of Mixed Land Use. Environment and Planning A: Economy and Space. 45**,** 2797-2798.

Ministry of Housing and Urban-Rural Development of the People's Republic of China, Code for classification of urban land use and planning standards of debbelopment land (GB 50137). 2011.

Sallis, J. F., et al., 2016. Physical activity in relation to urban environments in 14 cities worldwide: a cross-sectional study. Lancet. 387**,** 2207-2217.

Su, Y., et al., 2023. Measuring accessibility to health care using taxi trajectories data: a case study of acute myocardial infarction cases in Beijing. Int J Health Policy. 12**,** 6653.

WorldPop (<www.worldpop.org> - School of Geography and Environmental Science, University of Southampton; Department of Geography and Geosciences,, University of Louisville; Departement de Geographie,, Universite de Namur), Center for International Earth Science Information Network (CIESIN), Columbia University, Global High Resolution Population Denominators Project (Funded by The Bill and Melinda Gates Foundation (OPP1134076)). 2018.
